# DUSP7 regulates the activity of ERK2 to promote proper chromosome alignment during cell division

**DOI:** 10.1016/j.jbc.2021.100676

**Published:** 2021-04-16

**Authors:** Xiao Guo, Ivan Ramirez, Yenni A. Garcia, Erick F. Velasquez, Ankur A. Gholkar, Whitaker Cohn, Julian P. Whitelegge, Bobby Tofig, Robert Damoiseaux, Jorge Z. Torres

**Affiliations:** 1 Department of Chemistry and Biochemistry, University of California, Los Angeles, California, USA; 2 Pasarow Mass Spectrometry Laboratory, The Jane and Terry Semel Institute for Neuroscience and Human Behavior, David Geffen School of Medicine, University of California, Los Angeles, California, USA; 3 Molecular Biology Institute, University of California, Los Angeles, California, USA; 4 Jonsson Comprehensive Cancer Center, University of California, Los Angeles, California, USA; 5 California NanoSystems Institute, University of California, Los Angeles, California, USA; 6 Department of Molecular and Medical Pharmacology, University of California, Los Angeles, California, USA

**Keywords:** cell division, chromosome alignment, dual-specificity phosphatase 7 (DUSP7), extracellular signal-regulated kinase 2 (ERK2), mitogen-activated protein kinases (MAPK), mitosis, CT, C terminus, D7, DUSP7, DUSP, dual-specificity phosphatase, ERK, extracellular signal-regulated kinase, IF, immunofluorescence, IP, immunoprecipitation, KIM, kinase interaction motif, LAP, localization and affinity purification, NC, negative control, NT, N terminus, OE, overexpression, PI, phosphatase inhibitor, WT, wild type, Thy, thymidine

## Abstract

Human cell division is a highly regulated process that relies on the accurate capture and movement of chromosomes to the metaphase plate. Errors in the fidelity of chromosome congression and alignment can lead to improper chromosome segregation, which is correlated with aneuploidy and tumorigenesis. These processes are known to be regulated by extracellular signal-regulated kinase 2 (ERK2) in other species, but the role of ERK2 in mitosis in mammals remains unclear. Here, we have identified the dual-specificity phosphatase 7 (DUSP7), known to display selectivity for ERK2, as important in regulating chromosome alignment. During mitosis, DUSP7 bound to ERK2 and regulated the abundance of active phospho-ERK2 through its phosphatase activity. Overexpression of DUSP7, but not catalytically inactive mutants, led to a decrease in the levels of phospho-ERK2 and mitotic chromosome misalignment, while knockdown of DUSP7 also led to defective chromosome congression that resulted in a prolonged mitosis. Consistently, knockdown or chemical inhibition of ERK2 or chemical inhibition of the MEK kinase that phosphorylates ERK2 led to chromosome alignment defects. Our results support a model wherein MEK-mediated phosphorylation and DUSP7-mediated dephosphorylation regulate the levels of active phospho-ERK2 to promote proper cell division.

Critical to the fidelity of cell division is the accurate movement and alignment of chromosomes at the metaphase plate and their segregation during anaphase. Errors in these processes are linked to human developmental disorders and tumorigenesis (1). Previous research has underscored the importance of protein phosphorylation as a molecular switch to regulate the activity of cell division enzymes (2, 3). This is highlighted by the growing list of essential mitotic kinases and their substrates that carry out functions related to bipolar spindle assembly, kinetochore-microtubule attachment, chromosome congression, and chromosome segregation (4, 5, 6). Beyond well-established mitotic kinases, less studied phospho signaling pathways have been implicated in cell division including the Wnt, mTOR, and MAPK/ERK pathways, among which MAPK/ERK is phosphorylated by MEKs (mitogen-activated protein kinase or extracellular signal–regulated kinase kinase) to regulate downstream transcription factors (7, 8, 9). In *Xenopus laevis* ERK2 (extracellular signal–regulated kinase 2) is critical for the spindle assembly checkpoint (10, 11, 12). In mammalian cells ERK1/2 activity is necessary for the G1/S transition and early G2 events for timely entry into mitosis (13, 14). However, whether human ERK2 is active in mitosis and what roles it plays in human somatic cell division remains ambiguous.

Our RNAi screen for novel factors important for cell division identified the dual-specificity phosphatase 7 (DUSP7/MKP-X). DUSP7, DUSP6/MKP-3, and DUSP9/MKP-4 are members of the cytoplasmic ERK specific mitogen-activated protein kinase phosphatases (MKPs) subfamily that share similar amino acid sequences, subcellular localizations, and substrate preferences (15, 16, 17). DUSPs can dephosphorylate both tyrosine and serine/threonine residues and are important modulators of signaling pathways that regulate cellular processes such as proliferation and apoptosis (16, 17). DUSP7 exhibits selectivity toward ERK1/2 (18, 19, 20) and is a regulator of oocyte meiosis (21, 22, 23). DUSP7 contains an N terminal noncatalytic Rhodanese-like domain and a C-terminal dual-phosphatase domain. A conserved Kinase Interaction Motif (KIM) in the noncatalytic domain is essential for the interaction between MKPs and ERK (19, 24). Two key amino acid residues within the conserved catalytic sequence (H/V)C(*X*_5_)R(S/T) of the phosphatase domain, C331 and R337, are important for DUSP7’s phosphatase activity (25, 26). However, in contrast to MKPs such as DUSP6 and DUSP9, little is known about the physiological functions of DUSP7.

Here, we have determined that MEK phosphorylation activity and DUSP7 phosphatase activity regulate the levels of active phospho-ERK2, which is important for the fidelity of chromosome alignment and segregation during cell division.

## Results

### DUSP7 interacts with ERK2 and regulates the levels of phospho-ERK2

**Figure 1:**
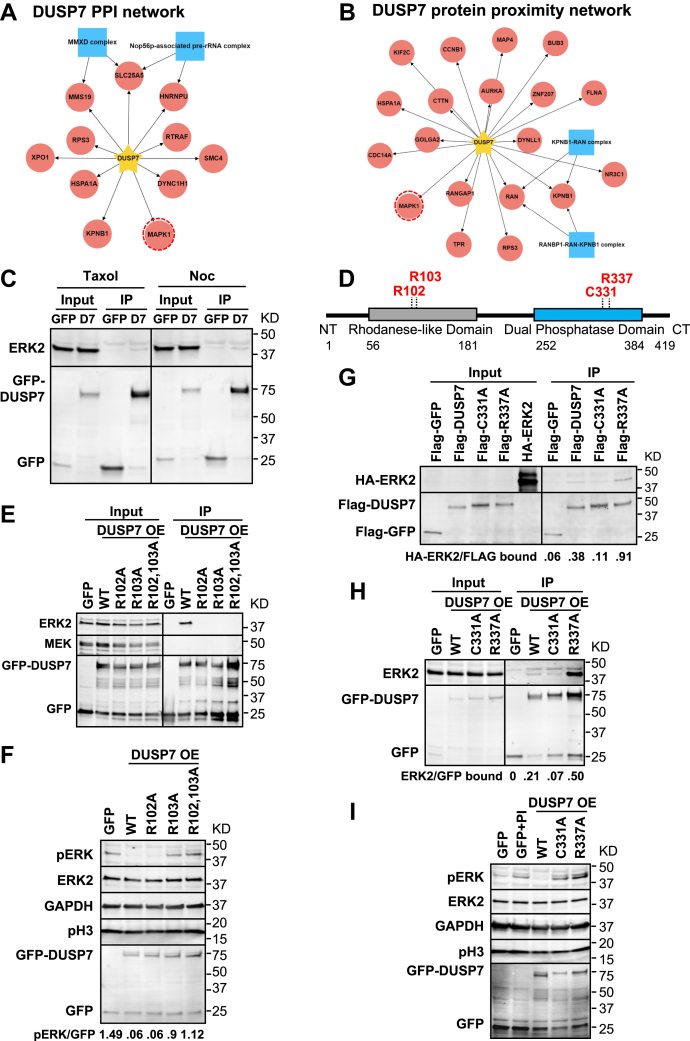
**DUSP7 interacts with ERK2 and regulates the levels of phospho-ERK2.***A* and *B*, DUSP7 protein–protein interaction (PPI) (*A*) and protein proximity (*B*) networks generated using mitotic spindle GO annotations and CORUM complex annotation analyses. *Yellow stars* indicate the bait protein DUSP7; *red circles* indicate putative interactors; *blue squares* indicate protein complexes; *red dashed circles* highlight ERK2 (aka MAPK1). *C*, ERK2 immunoprecipitates (IPs) with DUSP7 (D7) in early (nocodazole (Noc) arrested cells) and mid (taxol (Tax) arrested cells) mitosis. *D*, schematic of DUSP7 domain structure and key sites. The number of amino acids are indicated for each domain. DUSP7 KIM (R102, R103) and catalytic sites (C331, R337) are in *red*. *E*, the DUSP7 KIM mediates the DUSP7-ERK2 mitotic interaction. *F*, the DUSP7 KIM is dispensable for its phosphatase activity. Ratios below immunoblots indicate normalized phospho-ERK2 levels. *G* and *H*, the DUSP7-ERK2 mitotic interaction is influenced by DUSP7’s catalytic activity. In (*G*) HA-ERK2, Flag-DUSP7, Flag-C331A, Flag-R337A and Flag-GFP (negative control) were IVT expressed and incubated with anti-FLAG M2 magnetic beads in IP assays. In (*H*) LAP-only, LAP-DUSP7-WT, LAP-C331A, and LAP-R337A stable cell lines were induced before being harvested for S-tag pull downs. Ratios below immunoblots indicate relative protein–protein binding affinity. *I*, DUSP7 regulates mitotic phospho-ERK2 levels through its phosphatase activity. Phosphatase inhibitor (PI) in the second lane was added when lysing the cells. Numbers on the right side of immunoblots indicate molecular weights of proteins. All cell-based experiments and immunoprecipitations were carried out in HeLa cells.

To understand the role of DUSP7 during cell division, we began by defining the protein–protein interaction and protein proximity networks of DUSP7 in mitotic cells. Localization and affinity purification (LAP = GFP-Tev-S-tag)-tagged and biotin identification 2 (BioID2)-tagged DUSP7 inducible HeLa stable cell lines were used to express LAP/BioID2-DUSP7 and biochemical purifications were analyzed by mass spectrometry. In-house R scripts were used to analyze the mass spectrometry data and protein interaction and proximity networks were visualized with RCytoscape JS (Fig. S1, *A* and *B*). Further, we applied Gene Ontology (GO) terms (mitotic spindle; kinetochore and chromosome segregation) (Fig. S1*C*) and CORUM complex annotation analyses to these networks (see Experimental procedures for details). These analyses determined that ERK2 (aka MAPK1) was also associating with DUSP7 in mitosis (Fig. 1, *A* and *B*). Next, we validated the DUSP7-ERK2 mitotic interaction by immunoprecipitation (IP) experiments using mitotic cell extracts from Taxol- or nocodazole-arrested LAP-DUSP7 stable cell lines (Fig. 1*C*).

The KIM domain was shown to be essential for the interaction of some DUSPs (DUSP1, 4, 6) with ERK2 (24, 27, 28), but it remained unknown which domain of DUSP7 bound to ERK2 and whether its KIM was required for binding to ERK2 or its ability to dephosphorylate ERK2. To better understand the DUSP7-ERK2 interaction, we generated DUSP7 KIM mutants R102A, R103A, and R102,103A double mutants (Fig. 1*D* and Fig. S2, *A*–*C*). IP experiments from mitotic cells transiently transfected with DUSP7 or DUSP7 KIM mutants showed that ERK2 IPed with DUSP7 but not DUSP7 KIM mutants (Fig. 1*E*). To further define the interaction domains of DUSP7 involved in ERK2 binding, we generated a series of LAP-DUSP7 stable cell lines expressing DUSP7 truncations (Fig. S3*A*). ERK2 failed to associate with DUSP7 truncations (Fig. S3*B*), likely due to DUSP7 destabilization. Next, we sought to determine the significance of the DUSP7-ERK2 interaction. Consistent with the abolished interaction between DUSP7 KIM mutants and ERK2 (Fig. 1*E*), DUSP7-R103A and DUSP7-R102,103A double mutants showed a slightly reduced ability to dephosphorylate ERK2 in mitotic HeLa cells (Fig. 1*F* and Fig. S3, *C*–*E*). However, DUSP7-R102A could still dephosphorylate ERK2 (Fig. 1*F*); this phenomenon was also observed for conserved KIM mutations in DUSP6 (29). Similarly, IP experiments using *in vitro* expressed proteins or mitotic cell extracts from DUSP7 or DUSP7 catalytic dead mutant (C331A and R337A) (Fig. S2, *D*–*G*) cell lines showed that ERK2 IPed with DUSP7 and DUSP7-R337A but not DUSP7-C331A (Fig. 1, *G* and *H*, Fig. S3, *F*–*H*). While overexpression of DUSP7 led to the absence of phospho-ERK2, overexpressed DUSP7-R337A or DUSP7-C331A showed a reduced ability to dephosphorylate ERK2 in mitotic HeLa cells (Fig. 1*I*). Together, these results showed that DUSP7 was binding to ERK2 during mitosis and that the DUSP7 KIM was required for this interaction, while the DUSP7 catalytic sites (C331 and R337) within its phosphatase domain were important for regulating the levels of active phospho-ERK2.

### Knockdown of DUSP7 leads to chromosome alignment and segregation defects

**Figure 2:**
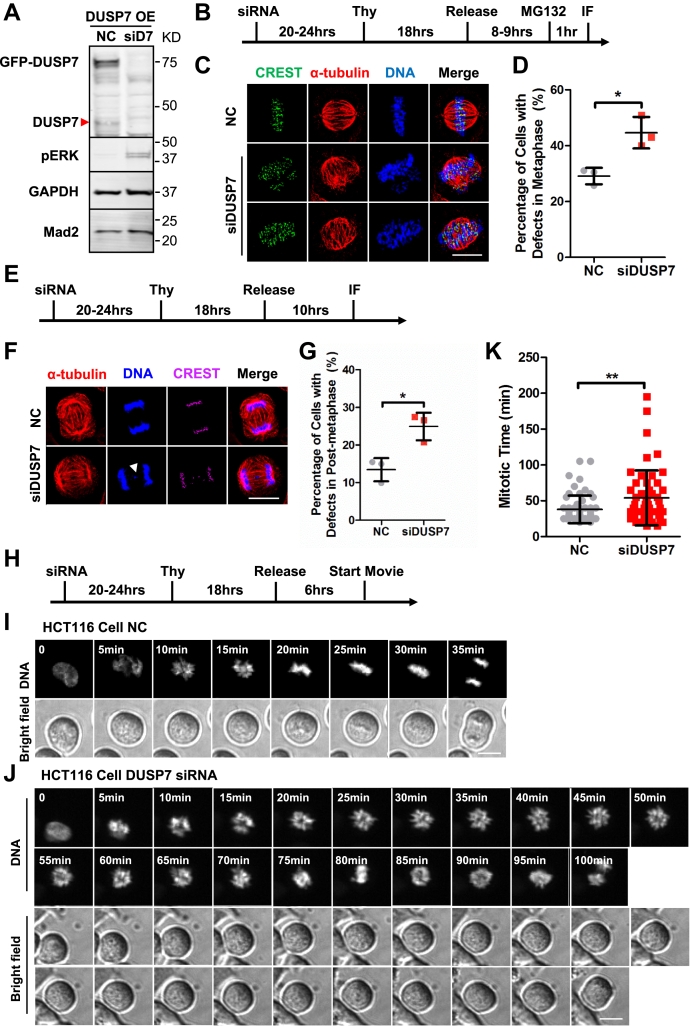
**Knockdown of DUSP7 leads to chromosome alignment and segregation defects.***A*, siRNA knockdown of endogenous and overexpressed DUSP7. Numbers on the right side of immunoblots indicate molecular weights of proteins. *Red arrow* indicates endogenous DUSP7 band. *B*, schematic of IF microscopy experiment performed in (*C*). *C*, knockdown of DUSP7 leads to chromosome misalignment in metaphase. HeLa cells were treated with negative control siRNA or siDUSP7 before being fixed and costained with anti-CREST and anti-α-tubulin antibodies and the DNA dye Hoechst 33342. *D*, quantification of the percentage of cells with chromosome misalignment in metaphase (y-axis) for conditions shown in (*C*) (x-axis). *E*, schematic of IF microscopy experiment performed in (*F*). *F*, knockdown of DUSP7 leads to an increase in lagging chromosomes in anaphase. HeLa cells were treated with negative control siRNA or siDUSP7 before being fixed and costained with anti-CREST and anti-α-tubulin antibodies and the DNA dye Hoechst 33342. *White arrow* shows the lagging chromosome. *G*, quantification of the percentage of cells with lagging chromosome in anaphase (y-axis) for conditions shown in (*F*) (x-axis). *H*, schematic of live-cell time-lapse microscopy experiment performed in (*I*) and (*J*). *I* and *J*, knockdown of DUSP7 leads to a slowed mitosis. Live-cell time-lapse microscopy of HCT116 GFP-H2B cells treated with negative control siRNA (*I*) and siDUSP7 (*J*) undergoing cell division. *K*, quantification of the timing of mitosis from chromosome condensation to chromosome segregation (y-axis) for the conditions shown in (*I*) and (*J*) (x-axis). Scale bars: 10 μm. ∗*p* < 0.05, ∗∗*p* < 0.01 (unpaired two-tailed Student’s *t*-test).

To understand the importance of DUSP7’s function in regulating the levels of active phospho-ERK2 during cell division, we first identified siRNAs capable of depleting DUSP7 levels by immunoblot analysis (Fig. 2*A* and Fig. S4*A*) and DUSP7 mRNA expression by RT-qPCR (Fig. S4, *B* and *C*). Knockdown of DUSP7 led to a failure to dephosphorylate ERK2 and an increase in phospho-ERK2 levels (Fig. 2*A*). We then analyzed the consequences of depleting DUSP7 during metaphase (Fig. 2*B*) and postmetaphase (Fig. 2*E*) with immunofluorescence (IF) microscopy. DUSP7 depletion led to an increased percentage of defective mitotic cells with chromosome misalignment (siDUSP7 = 44.6 ± 5.6, *p* < 0.05 compared with siControl = 29.1 ± 2.9) (Fig. 2, *C* and *D*). These defective cells also showed defects in spindle organization including unfocused and multipolar spindles (Fig. 2*C*). The chromosome misalignments defects in siDUSP7 cells translated into an increase in the percentage of lagging chromosomes during anaphase (siDUSP7 = 24.9 ± 3.7, *p* < 0.05 compared with siControl = 13.45 ± 3.1) (Fig. 2, *F* and *G*). The mitotic defects were rescued by an siRNA resistant DUSP7 (Fig. S2*H*) expressed at near endogenous levels but not DUSP7 catalytic dead mutants (Fig. S4, *D*–*F*). Similar results were observed and statistically analyzed in U2OS cells (Fig. S4, *G*–*I*) and HCT116 cells (Fig. S4, *J*–*L*).

Next, we analyzed whether DUSP7 depletion could affect the timing of cell division by live-cell time-lapse microscopy in HCT116 GFP-H2B cells (Fig. 2*H*). This analysis showed that depletion of DUSP7 led to a marked increase in the time from chromosome condensation to chromosome segregation (siDUSP7 = 54.0 ± 38.3 min, *p* < 0.01 compared with siControl = 38.0 ± 19.1 min) (Fig. 2, *I*–*K*; Movies S1–S4). Together, these results showed that depletion of DUSP7 led to a slowed mitosis where cells failed to properly align and segregate chromosomes.

### Downregulation of ERK2 leads to chromosome alignment defects

**Figure 3:**
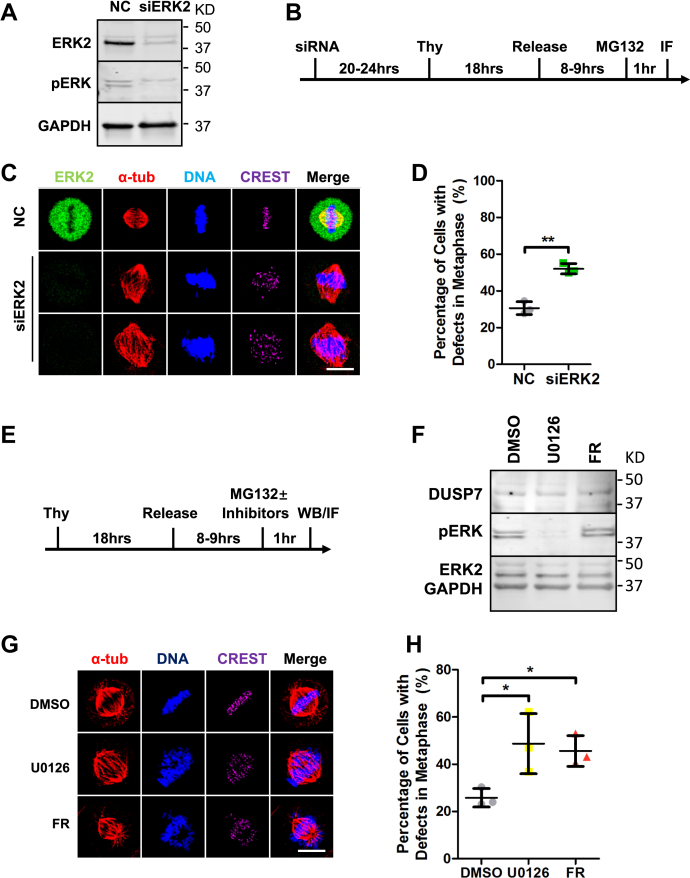
**Downregulation of ERK2 leads to chromosome alignment defects.***A*, siRNA knockdown of ERK2. *B*, schematic of IF microscopy experiment performed in (*C*). *C*, knockdown of ERK2 leads to chromosome misalignment in metaphase. HeLa cells were treated with negative control siRNA or siERK2 before being fixed and costained with anti-ERK2, anti-CREST and anti-α-tubulin antibodies and the DNA dye Hoechst 33342. *D*, quantification of the percentage of cells with chromosome misalignment in metaphase (y-axis) for conditions shown in (*C*) (x-axis). *E*, schematic of western blotting experiment performed in (*F*) and IF microscopy experiment performed in (*G*). *F*, HeLa cells were treated with DMSO (as negative control), 50 μM U0126, or 50 μM FR180204 before being lysed and analyzed by immunoblot. *G*, inhibition of MEK kinase activity or ERK2 kinase activity leads to chromosome misalignment in metaphase. HeLa cells were treated with DMSO or the indicated inhibitors, fixed, and costained with anti-CREST and anti-α-tubulin antibodies and the DNA dye Hoechst 33342. *H*, quantification of the percentage of cells with chromosome misalignment in metaphase (y-axis) for the conditions shown in (*G*) (x-axis). Numbers on the right side of immunoblots indicate molecular weights of proteins. Scale bars: 10 μm. ∗*p* < 0.05, ∗∗*p* < 0.01 (unpaired two-tailed Student’s *t*-test).

Next, we sought to determine if ERK2 was important for human cell division. First, we depleted endogenous ERK2 by RNAi (Fig. 3*A*) and analyzed the consequences during cell division (Fig. 3*B*) with IF microscopy. Depletion of ERK2 led to an increased number of cells with defects in chromosome alignment during metaphase (siERK2 = 52.1 ± 2.8, *p* < 0.01 compared with siControl = 30.6 ± 3.5) (Fig. 3, *C* and *D*), which was consistent in U2OS cells (Fig. S5, *A* and *B*) and HCT116 cells (Fig. S5, *C* and *D*). ERK2 depletion also led to an increase in interphase cells that were multinucleated or contained micronuclei (siERK2 = 22.6 ± 6.2, *p* < 0.01 compared with siControl = 3.8 ± 0.8) (Fig. S5, *E* and *F*).

Since phospho-ERK2 levels were lower in mitosis than in G1/S phase (Fig. S5, *G*–*J*), we asked if ERK2 phosphorylation or ERK2 kinase activity was important for cell division. HeLa cells were treated with a MEK inhibitor U0126 (30, 31) or the ERK2 ATP-competitive inhibitor FR180204 (32) and analyzed by western blotting and IF microscopy (Fig. 3*E*). Phospho-ERK2 levels decreased in U0126-treated cells, but were not affected in FR180204-treated cells (Fig. 3*F*). In comparison to the control DMSO treatment, cells treated with U0126 or FR180204 showed an increase in chromosome alignment errors (U0126 = 48.7 ± 12.7, *p* < 0.05 and FR180204 = 45.6 ± 6.5, *p* < 0.05 compared with DMSO = 25.8 ± 3.9) (Fig. 3, *G* and *H*), which was consistent in U2OS cells (Fig. S5, *K* and *L*) and HCT116 cells (Fig. S5, *M* and *N*). These results showed that inhibiting ERK2 phosphorylation, and thereby its activation, or ERK2’s kinase activity led to chromosome alignment defects.

### DUSP7 promotes chromosome alignment in mitosis by regulating the activity of ERK2

**Figure 4:**
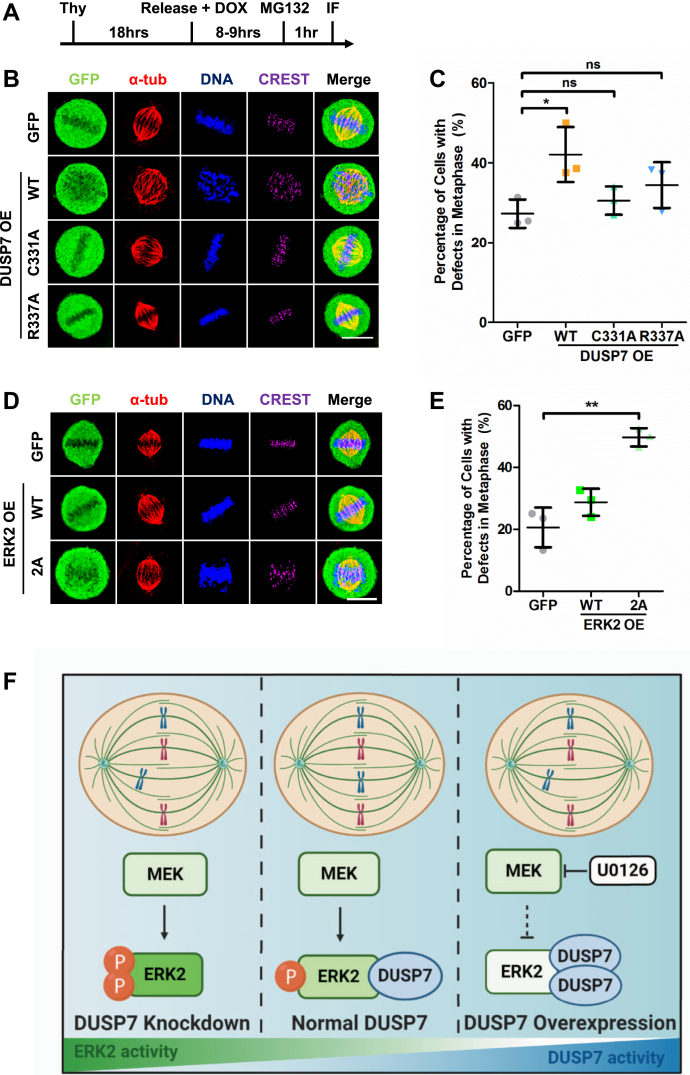
**DUSP7 promotes chromosome alignment in mitosis by regulating the activity of ERK2.***A*, schematic of IF microscopy experiment performed in (*B*) and (*D*). *B*, overexpression of DUSP7 wild type, but not catalytic dead mutants, leads to chromosome misalignment in metaphase. LAP-only, LAP-DUSP7-WT, LAP-C331A, and LAP-R337A HeLa stable cell lines were treated as described in (*A*) before being fixed and costained with anti-GFP, anti-CREST, and anti-α-tubulin antibodies and the DNA dye Hoechst 33342. *C*, quantification of the percentage of cells with chromosome misalignment in metaphase (y-axis) for conditions shown in (*B*) (x-axis). *D*, LAP-only, LAP-ERK2-WT, and LAP-ERK2-2A HeLa stable cell lines were treated as described in (*A*) before being fixed and costained with anti-GFP, anti-CREST, and anti-α-tubulin antibodies and the DNA dye Hoechst 33342. *E*, quantification of the percentage of cells with chromosome misalignment in metaphase (y-axis) for conditions shown in (*D*) (x-axis). *F*, model of how DUSP7 regulates the abundance of active phospho-ERK2 to ensure the fidelity of chromosome alignment. See main text for details. Scale bars: 10 μm. ∗*p* < 0.05, ∗∗*p* < 0.01, ns indicates not statistically significant (unpaired two-tailed Student’s *t*-test).

Since DUSP7 dephosphorylated ERK2 (Fig. 1*E*), we hypothesized that overexpression of DUSP7 would lead to similar chromosome alignment defects to those observed in cells treated with the MEK inhibitor U0126. To test this, we overexpressed GFP-tagged DUSP7 (validated to decrease phospho-ERK2 levels, Fig. 1, *F* and *I*) or the catalytic dead DUSP7-C331A or DUSP7-R337A mutants (showed minimal effects on phospho-ERK2 levels, Fig. 1*I*) and analyzed the cells by IF microscopy (Fig. 4*A*). While DUSP7 overexpression led to a significant increase in chromosome alignment defects, overexpression of DUSP7-R337A or DUSP7-C331A did not (DUSP7 = 42.1 ± 6.9, *p* < 0.05; DUSP7-C331A = 30.6 ± 3.5, *p* = 0.3183; and DUSP7-R337A = 34.4 ± 5.7, *p* = 0.1386; compared to the GFP control = 27.3 ± 3.5) (Fig. 4, *B* and *C*), which was consistent in U2OS cells (Fig. S6*A*) and HCT116 cells (Fig. S6*B*). These results showed that an overabundance of DUSP7 phosphatase activity led to chromosome alignment defects.

To further understand the phospho-ERK2 equilibrium regulated by MEK and DUSP7 during cell division, we asked if the ERK2-DUSP7 interaction was dependent on ERK2 phosphorylation. IP experiments using cell extracts from a U0126-treated LAP-DUSP7 stable cell line showed that ERK2 bound to DUSP7 in the absence of MEK kinase activity (Fig. S6*C*). Since ERK2 is phosphorylated by MEK at T185 and Y187 (33, 34), we generated the nonphosphorylation mimetic mutant ERK2-2A (T185,Y187A) (Fig. S2, *I* and *J*) and analyzed its binding to DUSP7. *In vitro* binding experiments showed that both ERK2 and ERK2-2A bound to DUSP7 (Fig. S6*D*). Similar results were observed in IP experiments from HeLa cell extracts (Fig. S6*E*). Together, these results showed that DUSP7’s binding to ERK2 did not require ERK2 to be phosphorylated. Instead, D318 within the ERK2 common docking (CD) domain (24) was responsible for its binding to DUSP7 (Fig. S6*F*). Next, we examined if phosphorylation of ERK2 at T185 and Y187 was critical for cell division by analyzing cells overexpressing GFP-tagged ERK2 or the nonphosphorylation mimetic mutant ERK2-2A (Fig. 4*A*). Compared with the overexpression of ERK2, overexpression of ERK2-2A led to a significant increase in cells with chromosome alignment defects in metaphase (ERK2 = 28.7 ± 4.4, *p* = 0.1438; ERK2-2A = 49.7 ± 3.0, *p* < 0.01; compared with GFP control = 20.6 ± 6.4) (Fig. 4, *D* and *E*), which was consistent in U2OS cells (Fig. S6*A*) and HCT116 cells (Fig. S6*B*). These data indicated that the proper amount of phospho-ERK2 in cells was critical for chromosome alignment and segregation during mitosis.

## Discussion

This study revealed a novel function for DUSP7 in mitotic chromosome alignment and established the MAPK/ERK pathway as being important for cell division. Our data are consistent with a model where, during a normal mitosis, MEK’s kinase activity phosphorylates ERK2 and DUSP7’s phosphatase activity dephosphorylates ERK2 to establish an equilibrium of active phospho-ERK2 (Fig. 4*F* middle panel). This phospho-ERK2 equilibrium is critical for ensuring the fidelity of chromosome alignment and segregation. Perturbing the balance of active phospho-ERK2 through MEK inhibition (Fig. 4*F* right panel), DUSP7 depletion (Fig. 4*F* left panel) or overexpression (Fig. 4*F* right panel) leads to defects in chromosome alignment. Together, these results establish DUSP7 as an important mitotic phosphatase that regulates the abundance of active phospho-ERK2 to ensure the fidelity of chromosome alignment and segregation.

Interestingly, although the DUSP7 KIM mutant R102A did not bind EKR2, it could still dephosphorylate it (Fig. 1*F*, Fig. S3, *C*–*E*). This is consistent with previous DUSP6 observations, where the DUSP6 KIM mutant R64A did not bind ERK2 but was able to dephosphorylate it (29). Therefore, it is possible that these mutants are capable of transiently interacting with ERK2, but that the interaction is undetectable in IP experiments.

With the exception of ERK2, there is little known about the repertoire of DUSP7 substrates, regulators, and interactors. The GO enrichment analyses of the DUSP7 protein association network and DUSP7 proximity protein network indicate that DUSP7 is likely to associate with numerous proteins that carry out important functions related to a broad array of cellular processes including apoptosis, regulation of transcription, and cell division (Table S1). Therefore, future studies aimed at understanding the importance of these interactions will further aid our understanding of DUSP7’s function in cell division and beyond.

## Experimental procedures

### Cell culture

Table S2 lists all reagents and tools used in this study. HeLa cells (ATCC) were grown in DMEM/Ham's F-12 with L-Glutamine (Genesee Scientific), U2OS (ATCC) and HCT116 cells were grown in McCoy's 5A (Gibco), with 10% FBS and 5% CO_2_ at 37 °C. Detailed experimental procedures for cell synchronization, cell transfection, and inhibitor treatments are in the Supporting information.

### Generation of vectors and cell lines

DUSP7 and ERK2 mutants were generated by QuikChange Lightning Site-Directed Mutagenesis (Agilent). cDNAs of GFP, DUSP7, DUSP7 KIM mutants, DUSP7 catalytic dead mutants, ERK2, ERK2-2A, and DUSP7 truncations were cloned into pGLAP1, pGBioID2, pCS2-HA, or pCS2-Flag *via* Gateway LR Clonase reaction (35). pGLAP1-only/DUSP7/DUSP7-C331A/DUSP7-R337A/ERK2/ERK2-2A/DUSP7-truncations and pGBioID2-only/DUSP7 were used to generate Dox inducible HeLa Flp-In T-REx LAP-GFP/DUSP7-C331A/DUSP7-R337A/ERK2/ERK2-2A/DUSP7-truncations and HeLa Flp-In T-REx BioID2-only/DUSP7 stable cell lines as described previously (36, 37) (see Supporting information).

### LAP/BioID2 purifications and LC-MS/MS analyses

LAP purifications from Taxol arrested LAP-tagged inducible stable cell lines were as previously described (36). For BioID2 purifications, biotinylated proteins were purified from Taxol-arrested BioID2-tagged inducible stable cell lines as described previously (38, 39). Mass spectrometry analysis was performed on a Thermo Q Exactive Plus Orbitrap as described previously (40). Protein–protein interaction information was integrated from the Biological General Repository for Interaction Datasets (BioGRID v. 3.5) (41). Protein-complex information was derived from the Comprehensive Resource of Mammalian Protein Complexes (CORUM v. 3.0) (42). Selected GO terms (Gene Ontology release June 2019) (43) were used to analyze the protein–protein interactions based on cellular mechanisms. Affinity-based and proximity-based networks were generated with RCytoscapeJS (44, 45). See Supporting information and Table S3–S6 for details on purifications, mass spectrometry, quantification of data, and protein interaction and proximity networks.

### Immunoprecipitations, *in vitro* binding assays, and immunoblot analyses

Immunoprecipitations, *in vitro* binding assays, and immunoblot analyses were performed as described previously (46) with minor modifications detailed in the Supporting information.

### Cell imaging

Fixed-cell and live-cell time-lapse microscopy was carried out as described previously (47), except that an ImageXpress XL imaging system (Molecular Devices) was used for live cell imaging. See Supporting information for details on imaging, quantification of data, and statistical analyses.

### RT-qPCR

RNA from control or DUSP7 siRNA transfected HeLa, U2OS, or HCT116 cells and DUSP7 cell lines was isolated with Direct-zol RNA Miniprep Kits (Zymo Research) and reverse transcribed with UltraScript 2.0 cDNA Synthesis Kit (Genesee Scientific). qPCR was carried out with the synthesized cDNA, oligo(dT) primers, and qPCRBIO SyGreen Blue Mix Lo-ROX (Genesee Scientific) using a CFX Connect Real-Time PCR Detection System (Bio-Rad). qPCR data were analyzed with the Livak–Schmittgen method (2^−ΔΔCT^) (48).

### Antibodies

See Table S2 for a list of the antibodies used for immunoblotting and IF microscopy.

## Data and code availability

Mass spectrometry data were deposited at the UCSD Center for Computational Mass Spectrometry MassIVE datasets ftp://massive.ucsd.edu/MSV000085629/. R scripts used to analyze and visualize LC-MS/MS results were deposited at GitHub https://github.com/uclatorreslab/MassSpecAnalysis. All remaining data are contained within this article.

## Supporting information

This article contains supporting information (46, 49, 50, 51).

## Conflict of interest

The authors declare that they have no conflicts of interest with the contents of this article.
