# Supplementary material for: DUSP7 regulates the activity of ERK2 to promote proper chromosome alignment during cell division
Source: J Biol Chem. 2021 Apr 16;296:100676. doi: 10.1016/j.jbc.2021.100676 (PMC8131738; doi:10.1016/j.jbc.2021.100676)
Supplement: Supplemental Figures S1–S6, Table S2 [file mmc3.pdf]

SUPPORTING INFORMATION

DUSP7 regulates the activity of ERK2 to promote proper chromosome alignment during cell division

Xiao Guo[1], Ivan Ramirez[1], Yenni A. Garcia[1], Erick F. Velasquez[1] , Ankur A. Gholkar[1], Whitaker Cohn[2], Julian P. Whitelegge[2,3,4], Bobby Tofig[5], Robert Damoiseaux[5,6] and Jorge Z. Torres[1, 3, 4*]

[1]Department of Chemistry and Biochemistry, University of California, Los Angeles, CA 90095, USA
[2]Pasarow Mass Spectrometry Laboratory, The Jane and Terry Semel Institute for Neuroscience and Human Behavior, David Geffen School of Medicine, University of California, Los Angeles, CA 90095, USA
[3]Molecular Biology Institute, University of California, Los Angeles, CA 90095, USA
[4]Jonsson Comprehensive Cancer Center, University of California, Los Angeles, CA 90095, USA
[5]California NanoSystems Institute, Los Angeles, CA 90095, USA
[6]Department of Molecular and Medical Pharmacology, Los Angeles, CA 90095, USA

*Corresponding author:
Jorge Z. Torres
607 Charles E. Young Drive East
Los Angeles, CA 90095
Phone: 310-206-2092
Fax: 310-206-5213
torres@chem.ucla.edu

Table of contents: **Page**

**SUPPORTING FIGURES**	**S2 – S12**
**SUPPORTING MOVIE LEGENDS**	**S12**
**SUPPORTING TABLE S1 LEGEND**	**S13**
**SUPPORTING TABLE S2**	**S13 – S18**
**SUPPORTING TABLE S3–S6 LEGENDS**	**S18**
**DETAILED EXPERIMENTAL PROCEDURES**	**S19 – S22**

SUPPORTING FIGURES

A **DUSP7 PPI network**

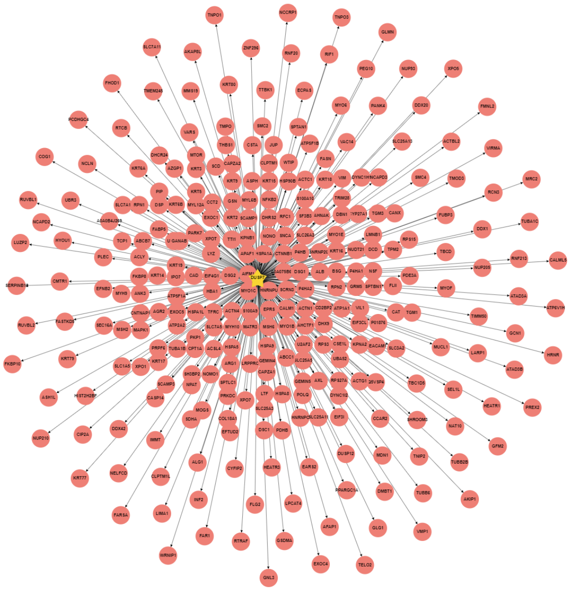

B **DUSP7 protein proximity network**

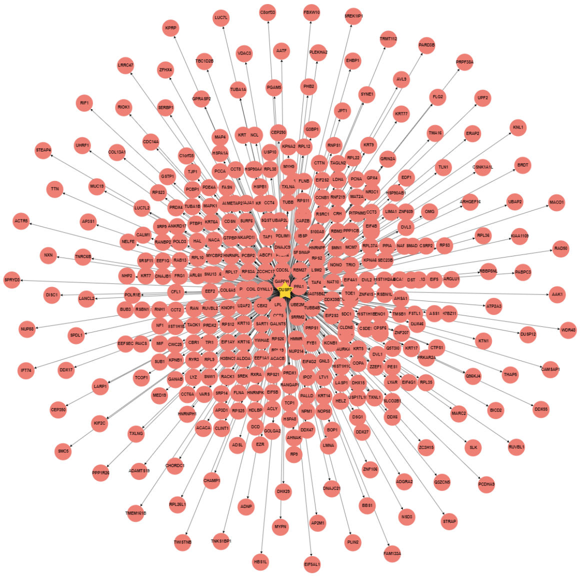

C

GO:ID	Ontology
GO:0000776	kinetochore
GO:0007079	mitotic chromosome movement towards spindle pole
GO:0007052	mitotic spindle organization
GO:1990023	mitotic spindle midzone
GO:0090307	mitotic spindle assembly
GO:0072686	mitotic spindle
GO:1901673	regulation of mitotic spindle assembly
GO:0040001	establishment of mitotic spindle localization
GO:0000070	mitotic sister chromatid segregation
GO:0007059	chromosome segregation

Figure S1. Mass spectrometry analysis of DUSP7 mitotic interactors. Related to Figure 1. **A.** DUSP7 protein-protein interaction (PPI) network comprised of proteins identified in LAP-DUSP7 tandem affinity purifications by mass spectrometry. **B.** DUSP7 protein proximity network comprised of proteins identified in BioID2-DUSP7 biotin purifications by mass spectrometry. **C.** GeneOntology (GO) terms used for generating PPI and protein proximity networks in Figure 1A and 1B.

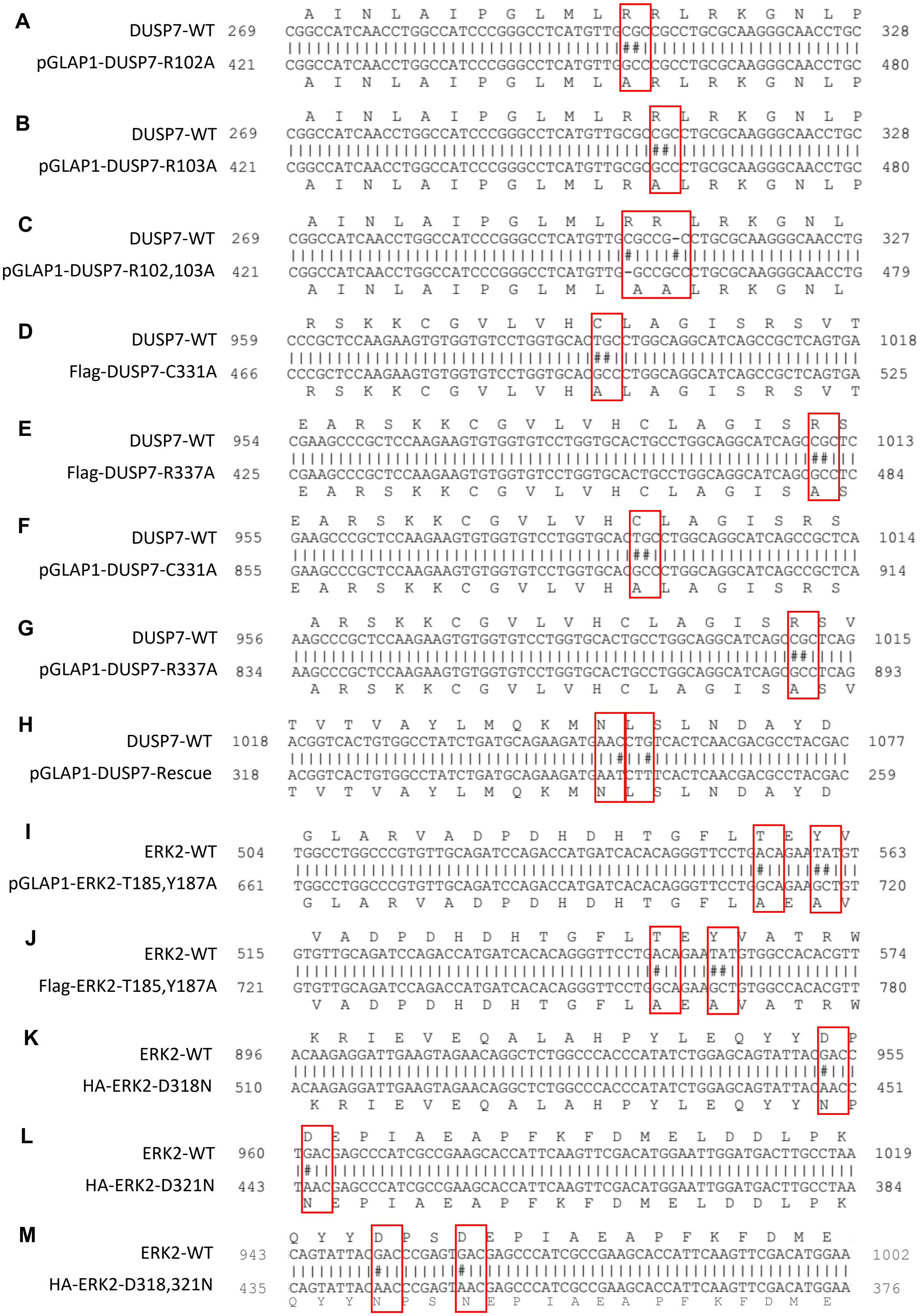
A
DUSP7-WT 269 328
pGLAP1-DUSP7-R102A 421 480
B
DUSP7-WT 269 328
pGLAP1-DUSP7-R103A 421 480
C
DUSP7-WT 269 327
pGLAP1-DUSP7-R102,103A 421 479
D
DUSP7-WT 959 1018
Flag-DUSP7-C331A 466 525
E
DUSP7-WT 954 1013
Flag-DUSP7-R337A 425 484
F
DUSP7-WT 955 1014
pGLAP1-DUSP7-C331A 855 914
G
DUSP7-WT 956 1015
pGLAP1-DUSP7-R337A 834 893
H
DUSP7-WT 1018 1077
pGLAP1-DUSP7-Rescue 318 259
I
ERK2-WT 504 563
pGLAP1-ERK2-T185,Y187A 661 720
J
ERK2-WT 515 574
Flag-ERK2-T185,Y187A 721 780
K
ERK2-WT 896 955
HA-ERK2-D318N 510 451
L
ERK2-WT 960 1019
HA-ERK2-D321N 443 384
M
ERK2-WT 943 1002
HA-ERK2-D318,321N 435 376

Figure S2. Verification of site-directed mutagenesis. Related to Figure 1, Figure 4, Figure S3, Figure S4, Figure S6. **A.** Sequencing data of pGLAP1-DUSP7-R102A showing successful mutation of DUSP7-R102 into alanine (highlighted in red box). **B.** Sequencing data of pGLAP1-DUSP7-R103A showing successful mutation of DUSP7-R103 into alanine (highlighted in red box). **C.** Sequencing data of pGLAP1-DUSP7-R102,103A showing successful mutation of DUSP7-R102,103 into alanine (highlighted in red box). **D.** Sequencing data of Flag-DUSP7-C331A showing successful mutation of DUSP7-C331 into alanine (highlighted in red box). **E.** Sequencing data of Flag-DUSP7-R337A showing successful mutation of DUSP7-R337 into alanine (highlighted in red box). **F.** Sequencing data of pGLAP1-DUSP7-C331A showing successful mutation of DUSP7-C331 into alanine (highlighted in red box). **G.** Sequencing data of pGLAP1-DUSP7-R337A showing successful mutation of DUSP7-R337 into alanine (highlighted in red box). **H.** Sequencing data of pGLAP1-DUSP7-Rescue showing successful mutation of DUSP7-WT into siRNA resistant mutant (highlighted in red boxes). **I.** Sequencing data of pGLAP1-ERK2-T185,Y187A showing successful mutation of ERK2-T185,Y187 into alanine (highlighted in red boxes). **J.** Sequencing data of Flag-ERK2-T185,Y187A showing successful mutation of ERK2-T185,Y187 into alanine (highlighted in red boxes). **K.** Sequencing data of HA-ERK2-D318N showing successful mutation of ERK2-D318 into asparagine (highlighted in red box). **L.** Sequencing data of HA-ERK2-D321N showing successful mutation of ERK2-D321 into asparagine (highlighted in red box). **M.** Sequencing data of HA-ERK2-D318,321N showing successful mutation of ERK2-D318,321 into asparagine (highlighted in red boxes).

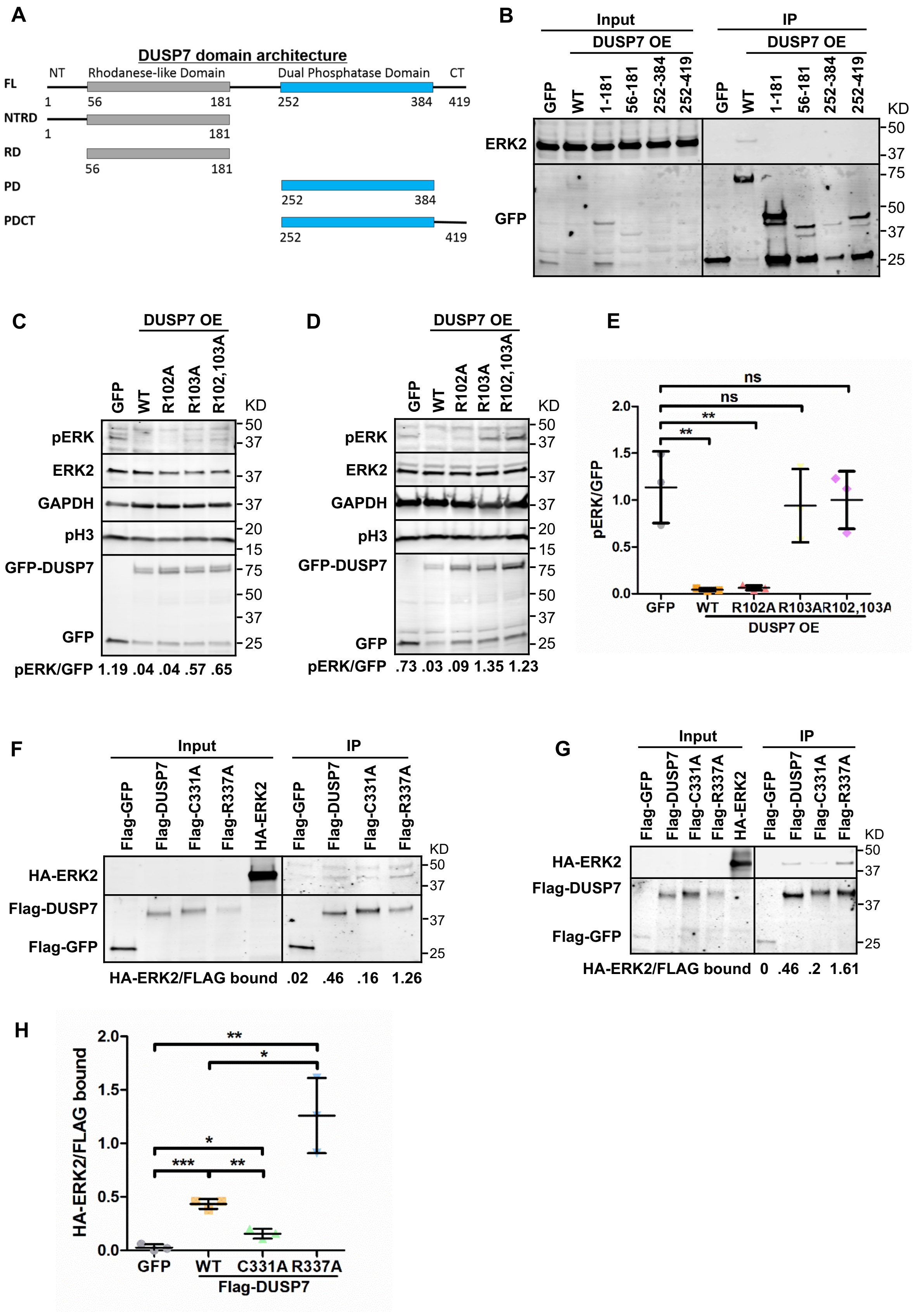
A
DUSP7 domain architecture
FL
NT
Rhodanese-like Domain
Dual Phosphatase Domain
CT
1 56 181 252 384 419
NTRD
1 181
RD
56 181
PD
252 384
PDCT
252 419
B
Input
IP
DUSP7 OE
GFP WT 1-181 56-181 252-384 252-419
KD
ERK2
GFP
50 37 75 50 37 25
C
DUSP7 OE
GFP WT R102A R103A R102,103A
KD
pERK
ERK2
GAPDH
pH3
GFP-DUSP7
GFP
pERK/GFP 1.19 .04 .04 .57 .65
D
DUSP7 OE
GFP WT R102A R103A R102,103A
pERK
ERK2
GAPDH
pH3
GFP-DUSP7
GFP
pERK/GFP .73 .03 .09 1.35 1.23
E
pERK/GFP
ns
ns
**
**
GFP WT R102A R103A R102,103A
DUSP7 OE
F
Input
IP
Flag-GFP Flag-DUSP7 Flag-C331A Flag-R337A HA-ERK2 Flag-GFP Flag-DUSP7 Flag-C331A Flag-R337A
KD
HA-ERK2
Flag-DUSP7
Flag-GFP
HA-ERK2/FLAG bound .02 .46 .16 1.26
G
Input
IP
Flag-GFP Flag-DUSP7 Flag-C331A Flag-R337A HA-ERK2 Flag-GFP Flag-DUSP7 Flag-C331A Flag-R337A
KD
HA-ERK2
Flag-DUSP7
Flag-GFP
HA-ERK2/FLAG bound 0 .46 .2 1.61
H
HA-ERK2/FLAG bound
GFP WT C331A R337A
Flag-DUSP7

Figure S3. DUSP7 interacts with and dephosphorylates ERK2. Related to Figure 1. **A.** Schematic of DUSP7 domain truncations. The numbers of amino acid residues are indicated for each domain truncation. Grey box represents Rhodanese-like domain, blue box represents dual phosphatase domain. FL=full length, NT=N terminus, CT=C terminus, NTRD=N terminus and Rhodanese-like domain, RD=Rhodanese-like domain, PD=phosphatase domain, PDCT=phosphatase domain and C terminus. **B.** ERK2 does not interact with DUSP7 truncations. LAP-only (GFP), LAP-DUSP7-WT and the indicated truncations were established in HeLa cells and their expression was induced with 0.1 μg ml^{-1} Doxycycline for 18 hours. Cells were harvested and protein extracts were used for S-tag pull downs, which were resolved by SDS PAGE, transferred to a PVDF membrane, and immunoblotted with the indicated antibodies. **C** and **D.** Triplication of Figure 1F. HeLa cells were transiently transfected with pGLAP1 only, pGLAP1-DUSP7-WT, or the pGLAP1-DUSP7-KIM mutants (R102A; R103A; R102A and R103A). Cells were arrested in mitosis with 100 nM Taxol for 18 hours before being harvested and cell lysates were resolved by SDS PAGE, transferred to a PVDF membrane, and immunoblotted with the indicated antibodies. Ratios below the immunoblots indicate the normalized phospho-ERK2 levels. **E.** Quantification of the normalized phospho-ERK2 level (y-axis) for the conditions shown in Figure 1F, Figure S3C and Figure S3D (x-axis). **F** and **G.** Triplication of Figure 1G. HA-ERK2, Flag-DUSP7, Flag-DUSP7-C331A, Flag-DUSP7-R337A, and Flag-GFP (negative control) were expressed in an IVT (*In Vitro* Transcription/Translation) system and incubated with anti-FLAG M2 magnetic beads in IP assays, which were resolved by SDS PAGE, transferred to a PVDF membrane, and immunoblotted with the indicated antibodies. Ratios below the immunoblots indicate the relative protein-protein binding affinity. **H.** Quantification of the relative protein-protein binding affinity (y-axis) for the conditions shown in Figure 1G, Figure S3F and S3G (x-axis).

Numbers on the right side of the western blots indicate the molecular weight of the proteins. Data were analyzed using unpaired two-tailed Student's t-test and represented as mean±s.d.; * indicates $P<0.05$, ** indicates $P<0.01$, *** indicates $P<0.001$, ns indicates not statistically significant. OE=overexpression, WT=wild type.

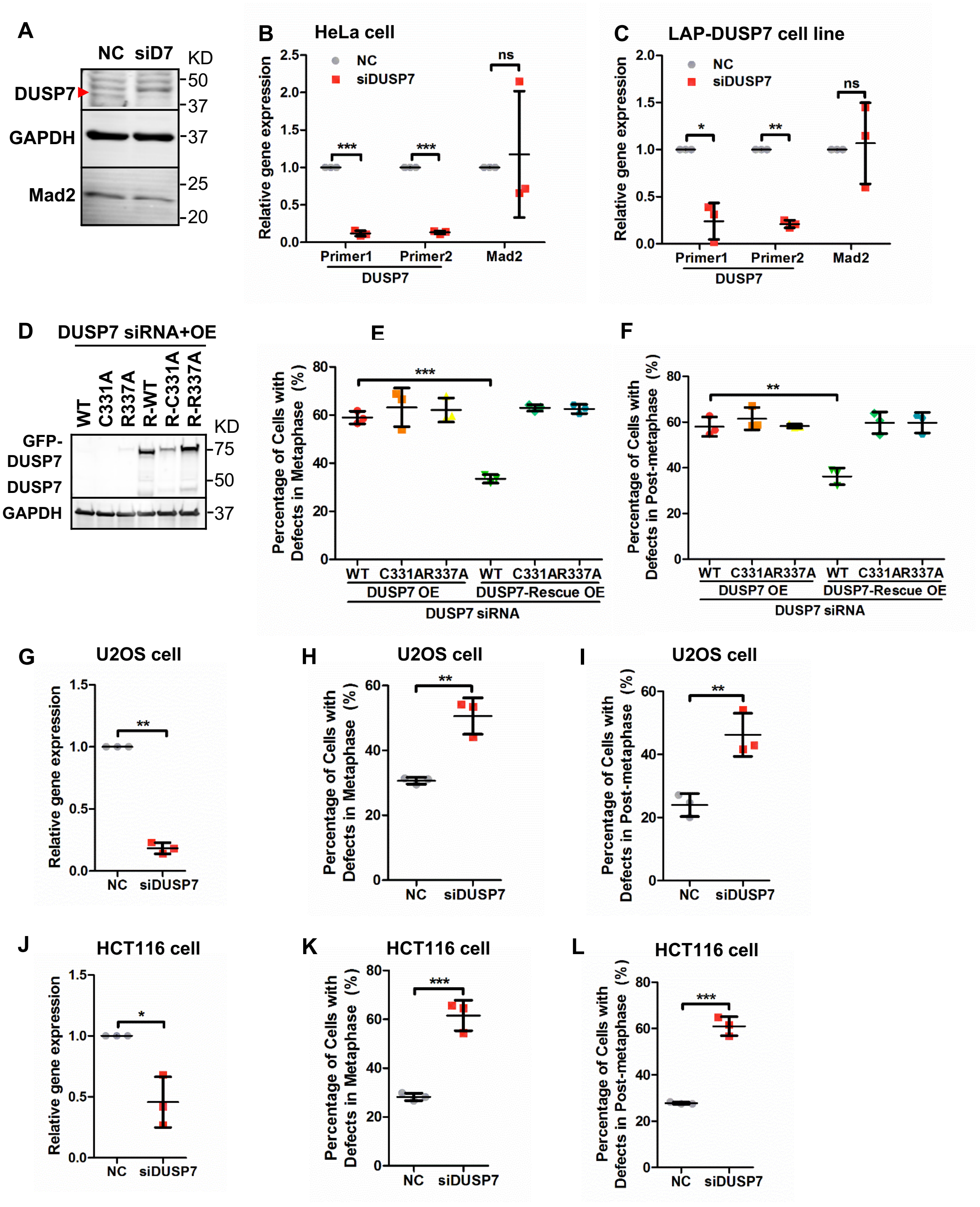
A
NC siD7
KD
DUSP7
GAPDH
Mad2
50
37
37
25
20
B HeLa cell
NC
siDUSP7
Relative gene expression
Primer1 Primer2 Mad2
DUSP7
C LAP-DUSP7 cell line
NC
siDUSP7
Relative gene expression
Primer1 Primer2 Mad2
DUSP7
D DUSP7 siRNA+OE
WT C331A R337A R-WT R-C331A R-R337A
KD
GFP-DUSP7
DUSP7
GAPDH
75
50
37
E
Percentage of Cells with Defects in Metaphase (%)
WT C331A R337A WT C331A R337A
DUSP7 OE
DUSP7-Rescue OE
DUSP7 siRNA
F
Percentage of Cells with Defects in Post-metaphase (%)
WT C331A R337A WT C331A R337A
DUSP7 OE
DUSP7-Rescue OE
DUSP7 siRNA
G U2OS cell
Relative gene expression
NC siDUSP7
H U2OS cell
Percentage of Cells with Defects in Metaphase (%)
NC siDUSP7
I U2OS cell
Percentage of Cells with Defects in Post-metaphase (%)
NC siDUSP7
J HCT116 cell
Relative gene expression
NC siDUSP7
K HCT116 cell
Percentage of Cells with Defects in Metaphase (%)
NC siDUSP7
L HCT116 cell
Percentage of Cells with Defects in Post-metaphase (%)
NC siDUSP7

Figure S4. Knockdown of DUSP7 leads to chromosome alignment and segregation defects. Related to Figure 2. **A.** siRNA knockdown of endogenous DUSP7. HeLa cells were transfected with control (NC) or DUSP7 siRNA (siD7) for 72 hours before being lysed and analyzed by immunoblot. Numbers on the right side of the immunoblots indicate the molecular weight of the proteins. Red arrow indicates endogenous DUSP7 band. **B** and **C.** siRNA knockdown of DUSP7 mRNA expression in HeLa cells. The relative gene expression of DUSP7 and Mad2 (as a negative control to show the siRNA specificity towards DUSP7) in HeLa cells (B) or LAP-DUSP7 cell lines (C) were normalized by GAPDH and analyzed with the Livak-Schmittgen method ($2^{-\Delta\Delta CT}$). Data represent the mean±s.d. of three independent experiments; technical triplicates were quantified for each experiment (n=9). **D.** DUSP7 rescue mutants are resistant to siRNA knockdown. HeLa cells were transfected with pGLAP1-DUSP7-WT, pGLAP1-DUSP7-C331A, pGLAP1-DUSP7-R337A and the corresponding siRNA resistant mutants post DUSP7 siRNA transfection. Cells were lysed and analyzed by immunoblot. Numbers on the right side of the immunoblots indicate the molecular weight of the proteins. **E.** Quantification of the percentage of cells with chromosome misalignment in metaphase (y-axis) for the conditions shown in (D) (x-axis). Three independent experiments were performed with 100 cells counted per experiment (n=300). Data were analyzed using unpaired two-tailed Student's t-test and represented as mean±s.d. **F.** Quantification of the percentage of cells with lagging chromosome in post-metaphase (y-axis) for the conditions shown in (D) (x-axis). Three independent experiments were performed with 100 cells counted per experiment (n=300). Data were analyzed using unpaired two-tailed Student's t-test and represented as mean±s.d. **G.** siRNA knockdown of DUSP7 mRNA expression in U2OS cells. The relative gene expression of DUSP7 in U2OS cells was normalized to GAPDH and analyzed with the Livak-Schmittgen method ($2^{-\Delta\Delta CT}$). Data represent the mean±s.d. of three independent experiments; technical triplicates were quantified for each experiment (n=9). **H** and **I.** Quantification of the percentage of U2OS cells with chromosome misalignment in metaphase (H) or defects in post-metaphase (I) (y-axis) for the conditions shown in (G) (x-axis). Three independent experiments were performed with 100 cells counted per experiment (n=300). Data were analyzed using unpaired two-tailed Student's t-test and represented as mean±s.d. **J.** siRNA knockdown of DUSP7 mRNA expression in HCT116 cells. The relative gene expression of DUSP7 in HCT116 cells was normalized to GAPDH and analyzed with the Livak-Schmittgen method ($2^{-\Delta\Delta CT}$). Data represent the mean±s.d. of three independent experiments; technical triplicates were quantified for each experiment (n=9). **K** and **L.** Quantification of the percentage of HCT116 cells with chromosome misalignment in metaphase (K) or defects in post-metaphase (L) (y-axis) for the conditions shown in (J) (x-axis). Three independent experiments were performed with 100 cells counted per experiment (n=300). Data were analyzed using unpaired two-tailed Student's t-test and represented as mean±s.d.
* indicates P<0.05, ** indicates P<0.01, *** indicates P<0.001, ns indicates not statistically significant (two-tailed Student's t-test). NC=negative control.

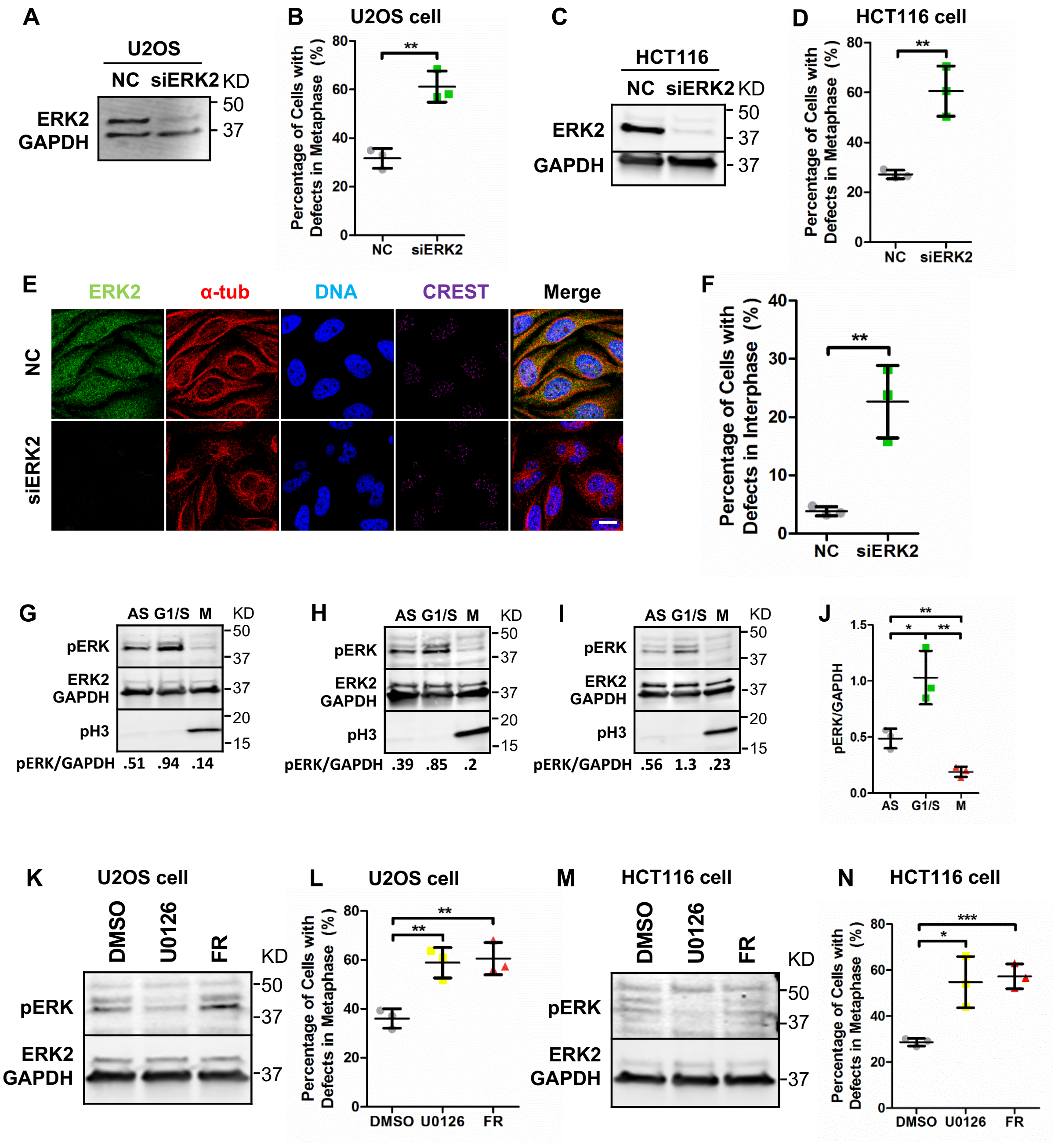

A
U2OS
NC siERK2 KD
ERK2
GAPDH
50
37
B
U2OS cell
Percentage of Cells with Defects in Metaphase (%)
**
NC siERK2
C
HCT116
NC siERK2 KD
ERK2
GAPDH
50
37
37
D
HCT116 cell
Percentage of Cells with Defects in Metaphase (%)
**
NC siERK2
E
ERK2
α-tub
DNA
CREST
Merge
NC
siERK2
F
Percentage of Cells with Defects in Interphase (%)
**
NC siERK2
G
AS G1/S M KD
pERK
ERK2
GAPDH
pH3
pERK/GAPDH .51 .94 .14
H
AS G1/S M KD
pERK
ERK2
GAPDH
pH3
pERK/GAPDH .39 .85 .2
I
AS G1/S M KD
pERK
ERK2
GAPDH
pH3
pERK/GAPDH .56 1.3 .23
J
pERK/GAPDH
AS G1/S M
K
U2OS cell
DMSO U0126 FR
KD
pERK
ERK2
GAPDH
L
U2OS cell
Percentage of Cells with Defects in Metaphase (%)
DMSO U0126 FR
M
HCT116 cell
DMSO U0126 FR
KD
pERK
ERK2
GAPDH
N
HCT116 cell
Percentage of Cells with Defects in Metaphase (%)
DMSO U0126 FR

Figure S5. Knockdown of ERK2 leads to cell division defects. Related to Figure 3. **A.** siRNA knockdown of endogenous ERK2 in U2OS cells. U2OS cells were transfected with negative control (NC) or ERK2 siRNA (siERK2) for 72 hours before being lysed and analyzed by immunoblot. **B.** Quantification of the percentage of U2OS cells with chromosome misalignment in metaphase (y-axis) for the conditions shown in (A) (x-axis). Three independent experiments were performed with 100 cells counted per experiment (n=300). **C.** siRNA knockdown of endogenous ERK2 in HCT116 cells. HCT116 cells were transfected with negative control (NC) or ERK2 siRNA (siERK2) for 72 hours before being lysed and analyzed by immunoblot. **D.** Quantification of the percentage of HCT116 cells with chromosome misalignment in metaphase (y-axis) for the conditions shown in (C) (x-axis). Three independent experiments were performed with 100 cells counted per experiment (n=300). **E.** Knockdown of ERK2 leads to defects during interphase. HeLa cells were treated with negative control siRNA (NC) or siERK2 before being fixed and co-stained with anti-ERK2, anti-CREST and anti-α-tubulin antibodies and the DNA dye Hoechst 33342. Scale bar: 10 μm. **F.** Quantification of the percentage of cells with multi-nuclei or micronuclei in interphase (y-axis) for the conditions shown in (E) (x-axis). Three independent experiments were performed with 100 cells counted per experiment (n=300). **G–I.** Phospho-ERK2 levels are lower in mitosis than in G1/S phase. HeLa cells were arrested in G1/S phase with 2 mM thymidine or in mitosis with 100 nM Taxol for 18 hours before being lysed and analyzed by immunoblot. Ratios below the immunoblots indicate the normalized phospho-ERK2 levels. **J.** Quantification of the normalized phospho-ERK2 levels (y-axis) for the conditions shown in (G–I) (x-axis). **K.** U2OS cells were treated with DMSO (as negative control), 50 μM U0126 or 50 μM FR180204 for 1 hour before being lysed and analyzed by immunoblot. **L.** Quantification of the percentage of U2OS cells with chromosome misalignment in metaphase (y-axis) for the conditions shown in (K) (x-axis). Three independent experiments were performed with 100 cells counted per experiment (n=300). **M.** HCT116 cells were treated with DMSO (as negative control), 50 μM U0126 or 50 μM FR180204 for 1 hour before being lysed and analyzed by immunoblot. **N.** Quantification of the percentage of HCT116 cells with chromosome misalignment in metaphase (y-axis) for the conditions shown in (M) (x-axis). Three independent experiments were performed with 100 cells counted per experiment (n=300).
Numbers on the right side of the immunoblots indicate the molecular weight of the proteins. Data were analyzed using unpaired two-tailed Student's t-test and represented as mean±s.d.; * indicates $P<0.05$, ** indicates $P<0.01$, *** indicates $P<0.001$.

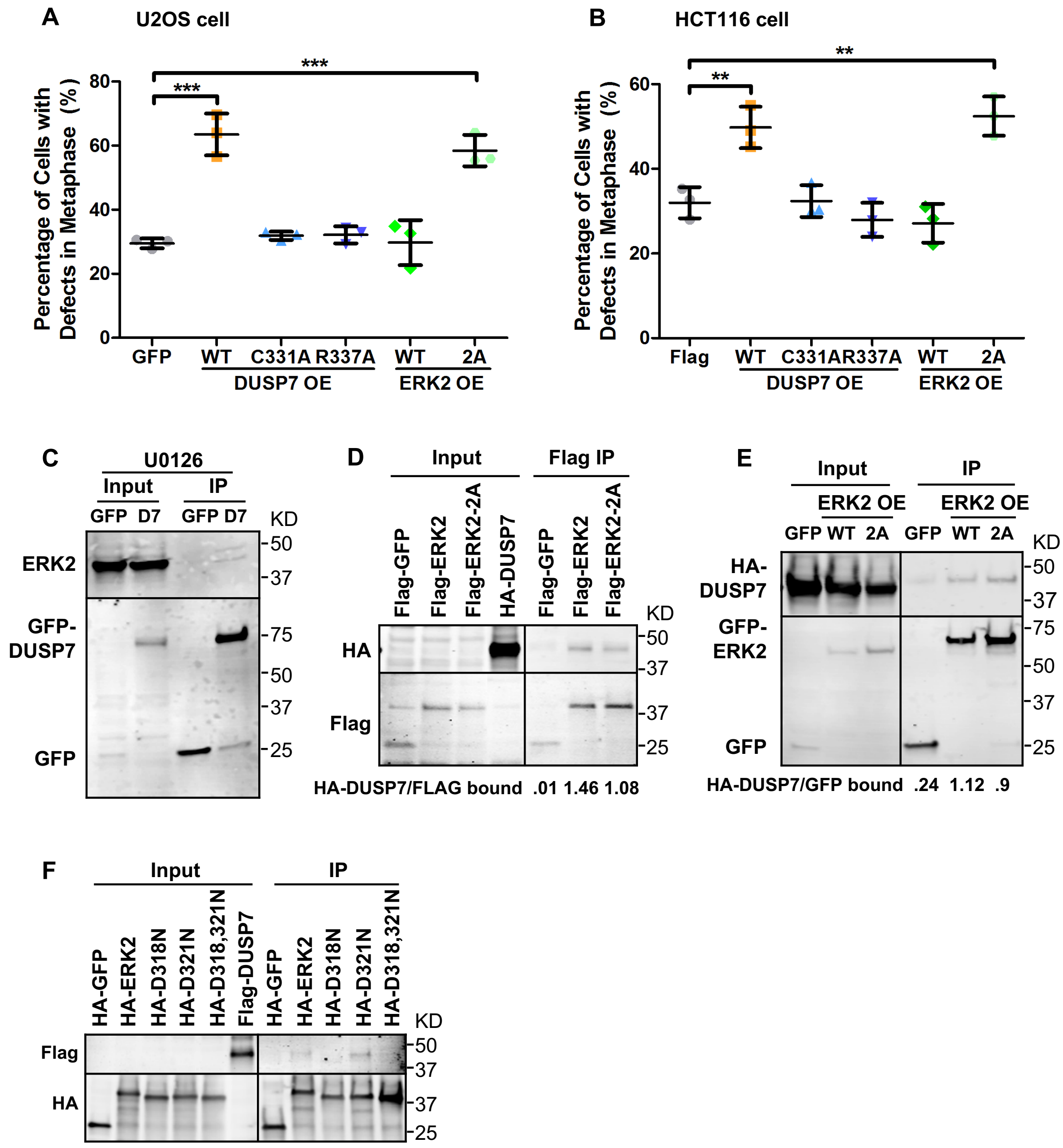
A
U2OS cell
Percentage of Cells with Defects in Metaphase (%)
GFP
WT
C331A
R337A
WT
2A
DUSP7 OE
ERK2 OE
B
HCT116 cell
Flag
C
U0126
Input
IP
GFP D7 GFP D7
ERK2
GFP-DUSP7
GFP
D
Flag IP
Flag-GFP
Flag-ERK2
Flag-ERK2-2A
HA-DUSP7
HA
Flag
HA-DUSP7/FLAG bound .01 1.46 1.08
E
GFP-ERK2
HA-DUSP7/GFP bound .24 1.12 .9
F
HA-GFP
HA-ERK2
HA-D318N
HA-D321N
HA-D318,321N
Flag-DUSP7
KD

Figure S6. DUSP7 promotes chromosome alignment in mitosis by regulating the activity of ERK2. Related to Figure 4. **A.** Quantification of the percentage of U2OS cells with chromosome misalignment in metaphase (y-axis) for the conditions shown in Figure 4B and 4D (x-axis). Three independent experiments were performed with 100 cells counted per experiment (n=300). Data were analyzed using unpaired two-tailed Student's t-test and represented as mean±s.d.; *** indicates P<0.001. **B.** Quantification of the percentage of HCT116 cells with chromosome misalignment in metaphase (y-axis) for the conditions shown in Figure 4B and 4D (x-axis). Three independent experiments were performed with 100 cells counted per experiment (n=300). Data were analyzed using unpaired two-tailed Student's t-test and represented as mean±s.d.; ** indicates P<0.01. **C.** ERK2 binds to DUSP7 in the presence of MEK kinase inhibitors. LAP-only and LAP-DUSP7-WT HeLa stable cell lines were induced with 0.1 µg ml-1 Doxycycline and inhibited with 50 µM U0126 for 18 hours before being harvested for S-tag pull downs. Pull downs were resolved by SDS PAGE, transferred to a PVDF membrane, and immunoblotted with the indicated antibodies. **D.** Non-phosphorylatable ERK2 mutant binds to DUSP7 *in vitro*. HA-DUSP7, Flag-ERK2, Flag-ERK2-2A (non-phosphorylatable mutant) and Flag-GFP (negative control) were expressed in an IVT (*In Vitro* Transcription/Translation) system and incubated with anti-FLAG M2 magnetic beads in immunoprecipitation assays. IPs were resolved by SDS PAGE, transferred to a PVDF membrane, and immunoblotted with indicated antibodies. **E.** Non-phosphorylatable ERK2 mutant binds to DUSP7 in cell extracts. LAP-only, LAP-ERK2-WT and LAP-ERK2-2A HeLa stable cell lines were transiently transfected with HA-DUSP7 and induced by 0.1 µg ml^{-1} Doxycycline for 18 hours before being harvested for S-tag beads pull down. Pull downs were resolved by SDS PAGE, transferred to a PVDF membrane, and immunoblotted with indicated antibodies. **F.** DUSP7 binds to ERK2 at D318 within its common docking (CD) domain. Flag-DUSP7, HA-GFP (negative control), HA-ERK2 and HA-ERK2 CD domain mutants (D318N; D321N; D318N and D321N) were expressed in an IVT (*In Vitro* Transcription/Translation) system and incubated with anti-HA magnetic beads in immunoprecipitation assays. IPs were resolved by SDS PAGE, transferred to a PVDF membrane, and immunoblotted with indicated antibodies.
Numbers on the right side of the immunoblots indicate the molecular weight of the proteins. Ratios below the immunoblots (D) and (E) indicate the relative protein-protein binding affinity. OE=overexpression, D7=DUSP7, 2A=T185,Y187A, WT=wild type.

SUPPORTING MOVIE LEGENDS

Movies S1–S2. Live-cell time-lapse microscopy movie of a representative siControl treated HCT116 GFP-H2B cell undergoing cell division. Related to Figure 2. HCT116 GFP-H2B cells were transfected with control siRNA, treated as described in Figure 2H and imaged live six hours post thymidine release for 18 hours with an ImageXpress XL imaging system at 37°C in 5% CO_2 using a 20x air objective. Images were captured every 5 minutes with bright field (Movie S1) and GFP (Movie S2) channels and converted to AVI movies with ImageJ at one frame per second. Each frame represents a five-minute interval.

Movies S3–S4. Live-cell time-lapse microscopy movie of representative siDUSP7 treated HCT116 GFP-H2B cell undergoing cell division. Related to Figure 2. HCT116 GFP-H2B cells were transfected with DUSP7 siRNA, treated as described in Figure 2H, and imaged live six hours post thymidine release for 18 hours with an ImageXpress XL imaging system at 37°C in 5% CO_2 using a 20x air objective. Images were captured every five minutes with bright field (Movie S3) and GFP (Movie S4) channels and converted to AVI movies with ImageJ at one frame per second. Each frame represents a five-minute interval.

SUPPORTING TABLE LEGENDS

Table S1. List of Gene Ontology (GO) annotations identified in the GO enrichment analysis of the DUSP7 interacting protein network and protein proximity association network analyses. List of GO terms identified in the GO enrichment analysis with Biological Process category, including GO term ID, GO annotation, gene related to the identified GO term, and the frequency of the GO terms identified in the GO enrichment analysis.

Table S2. List of key reagents and resources used in this study.

REAGENT or RESOURCE	SOURCE	IDENTIFIER
Antibodies		
Mouse monoclonal anti-ERK2 (D-2)	Santa Cruz Biotechnology	Cat# sc-1647; RRID: AB_627547
Chicken polyclonal anti-GFP	Abcam	Cat# ab13970; RRID: AB_300798
Mouse monoclonal anti-MEK-1 (H-8)	Santa Cruz Biotechnology	Cat# sc-6250; RRID: AB_627922
Rabbit polyclonal anti-HA	Proteintech	Cat# 51064-2-AP; RRID: AB_11042321
Mouse monoclonal anti-FLAG Dylight 800 Conjugated (clone 29E4.G7)	Rockland	Cat# 200-345-383; RRID: AB_10702994
Rabbit monoclonal anti-phospho-p44/42 MAPK (Erk1/2) (Thr202/Tyr204) (clone D13.14.4E)	Cell Signaling Technology	Cat# 4370; RRID: AB_2315112
Mouse monoclonal anti-GAPDH (clone 1E6D9)	Proteintech	Cat# 60004-1-Ig; RRID: AB_2107436
Rabbit polyclonal anti-phospho-Histone H3 (Ser10)	Millipore	Cat# 06-570; RRID: AB_310177
Rabbit polyclonal anti-DUSP7/PYST2	Proteintech	Cat# 26910-1-AP
Goat polyclonal anti-Mad2 (C-19)	Santa Cruz Biotechnology	Cat# sc-6329; RRID: AB_648599
Human polyclonal anti-Centromere Protein/CREST	Antibodies Incorporated	Cat# 15-234-0001; RRID: AB_2687472
Rat monoclonal anti-α-Tubulin (clone YOL1/34)	Bio-Rad	Cat# MCA78G; RRID: AB_325005
Donkey polyclonal anti-Human IgG (H+L), Fluorescein (FITC) AffiniPure	Jackson ImmunoResearch Labs	Cat# 709-095-149; RRID: AB_2340514
Donkey polyclonal anti-Rat IgG (H+L), Cy3 AffiniPure	Jackson ImmunoResearch Labs	Cat# 712-165-153; RRID: AB_2340667
Donkey polyclonal anti-Human IgG (H+L), Cy5 AffiniPure	Jackson ImmunoResearch Labs	Cat# 709-175-149; RRID: AB_2340539
Donkey polyclonal anti-Chicken IgY (IgG) (H+L), Fluorescein (FITC) AffiniPure	Jackson ImmunoResearch Labs	Cat# 703-095-155; RRID: AB_2340356
Donkey polyclonal anti-Mouse IgG (H+L), Fluorescein (FITC) AffiniPure	Jackson ImmunoResearch Labs	Cat# 715-095-151; RRID: AB_2335588
Donkey polyclonal anti-Goat IgG (H+L), IRDye 680RD	LI-COR Biosciences	Cat# 926-68074; RRID: AB_10956736

Donkey polyclonal anti-Mouse IgG (H+L), IRDye 680RD	LI-COR Biosciences	Cat# 926-68072; RRID: AB_10953628
Donkey polyclonal anti-Mouse IgG (H+L), IRDye 800CW	LI-COR Biosciences	Cat# 926-32212; RRID: AB_621847
Donkey polyclonal anti-Chicken IgG (H+L), IRDye 800CW	LI-COR Biosciences	Cat# 926-32218; RRID: AB_1850023
Donkey polyclonal anti-Rabbit IgG (H+L), IRDye 680RD	LI-COR Biosciences	Cat# 926-68073; RRID: AB_10954442
Donkey polyclonal anti-Rabbit IgG (H+L), IRDye 800CW	LI-COR Biosciences	Cat# 926-32213; RRID: AB_621848
Chemicals, Peptides, and Recombinant Proteins		
U0126	Selleckchem	Cat# S1102; CAS: 1173097-76-1
FR 180204	Selleckchem	Cat# S7524; CAS: 865362-74-9
Paclitaxel	Sigma-Aldrich	Cat# T7191; CAS: 33069-62-4
Nocodazole	Sigma-Aldrich	Cat# 1404; CAS: 31430-18-9
Thymidine	Sigma-Aldrich	Cat# T1895; CAS: 50-89-5
Hygromycin B	Thermo Fisher Scientific	Cat# 10687010
Doxycycline	Sigma-Aldrich	Cat# D9891; CAS: 24390-14-5
MG132	Millipore Sigma	Cat# 474790; CAS: 133407-82-6
Halt Protease Inhibitor Cocktail	Thermo Fisher Scientific	Cat# 87786
Phosphatase Inhibitor Cocktail 2	Sigma-Aldrich	P5726
Phosphatase Inhibitor Cocktail 3	Sigma-Aldrich	P0044
Hoechst 33342	Thermo Fisher Scientific	Cat# H1399; CAS: 23491-52-3
ProLong Gold Antifade Mountant	Thermo Fisher Scientific	Cat# P36934
Lipofectamine RNAiMAX	Thermo Fisher Scientific	Cat# 13778150
FuGENE HD Transfection Reagent	Promega	Cat# E2311
FuGENE 6 Transfection Reagent	Promega	Cat# E2691
S-protein Agarose	Millipore Sigma	Cat# 69704-4
Anti-FLAG M2 magnetic beads	Sigma-Aldrich	Cat# M8823
Anti-HA-tag mAb-magnetic beads	MBL International	Cat# M132-11
Biotin	Sigma-Aldrich	Cat# B4501-1G
Dynabeads MyOne Streptavidin C1	Thermo Fisher Scientific	Cat# 65002
Critical Commercial Assays		
Gateway LR Clonase II Enzyme mix	Thermo Fisher Scientific	Cat# 11791020
Gateway BP Clonase II Enzyme mix	Thermo Fisher Scientific	Cat# 11789020
QuikChange Lightning Site-Directed Mutagenesis Kit	Agilent	Cat# 210518
SP6 TnT Quick Coupled Transcription/Translation System	Promega	Cat# L2080
PureYield Plasmid Miniprep System	Promega	Cat# A1222

QIAprep Spin Miniprep Kit	QIAGEN	Cat# 27106
PureYield Plasmid Midiprep System	Promega	Cat# A2495
Direct-zol RNA Miniprep Kits	Zymo Research	Cat# R2051
UltraScript 2.0 cDNA Synthesis Kit	Genesee Scientific	Cat# 17-702
qPCRBIO SyGreen Blue Mix Lo-ROX	Genesee Scientific	Cat# 17-505
Deposited Data		
Affinity-based mass spectrometry performed with LAP-DUSP7	This paper	ftp://massive.ucsd.edu/MSV000085629/
Proximity-based mass spectrometry performed with BioID2-DUSP7	This paper	ftp://massive.ucsd.edu/MSV000085629/
Experimental Models: Cell Lines		
HeLa cell line	ATCC	Cat# CCL-2;<br>RRID: CVCL_0030
U2OS cell line	ATCC	Cat# HTB-96<br>PRID: CVCL_0042
HCT116 cells constitutively expressing GFP-H2B	This paper	N/A
HeLa Flp-In T-Rex cell lines	Stephen Taylor Lab	(49)
Inducible HeLa LAP-DUSP7 stable cell line	This paper	N/A
Inducible HeLa BioID2-DUSP7 stable cell line	This paper	N/A
Inducible HeLa LAP-DUSP7-C331A stable cell line	This paper	N/A
Inducible HeLa LAP-DUSP7-R337A stable cell line	This paper	N/A
Inducible HeLa LAP-DUSP7-1-181aa stable cell line	This paper	N/A
Inducible HeLa LAP-DUSP7-56-181aa stable cell line	This paper	N/A
Inducible HeLa LAP-DUSP7-252-384aa stable cell line	This paper	N/A
Inducible HeLa LAP-DUSP7-252-419aa stable cell line	This paper	N/A
Inducible HeLa LAP-ERK2 stable cell line	This paper	N/A
Inducible HeLa LAP-ERK2-2A (T185,Y187A) stable cell line	This paper	N/A
Oligonucleotides		
siRNA targeting DUSP7	Thermo Fisher Scientific	Cat# 4390824; siRNA ID: s4381
siRNA targeting ERK2	Thermo Fisher Scientific	Cat#: 4427038; siRNA ID: s11138
Primer1 for DUSP7 qPCR: Fwd 5'-GACGTGCTCGGCAAGTATG-3'	Eurofins Genomics	N/A
Primer1 for DUSP7 qPCR: Rev 5'-GGATCTGCTTGTAGGTGAACTC-3'	Eurofins Genomics	N/A
Primer2 for qPCR: Fwd 5'-TGTGGCCTATCTGATGCAGAA-3'	Eurofins Genomics	N/A
Primer2 for qPCR: Rev 5'-GGGCGAGATGTTGGACTTTTTC-3'	Eurofins Genomics	N/A

Primer for cloning DUSP7-R102A: Fwd 5'-CTTGCGCAGGCGGGCCAACATGAGGCCCG-3'	Eurofins Genomics	N/A
Primer for cloning DUSP7-R102A: Rev 5'-CGGGCCTCATGTTGGCCCGCCTGCGCAAG-3'	Eurofins Genomics	N/A
Primer for cloning DUSP7-R103A: Fwd 5'-GCCCTTGCGCAGGGCGCGCAACATGAGG-3'	Eurofins Genomics	N/A
Primer for cloning DUSP7-R103A: Rev 5'-CCTCATGTTGCGCGCCCTGCGCAAGGGC-3'	Eurofins Genomics	N/A
Primer for cloning DUSP7-R102,103A: Fwd 5'-TGCCCTTGCGCAGGGCGGCCAACATGAGGCCCG-3'	Eurofins Genomics	N/A
Primer for cloning DUSP7-R102,103A: Rev 5'-CGGGCCTCATGTTGGCCGCCCTGCGCAAGGGCA-3'	Eurofins Genomics	N/A
Primer for cloning DUSP7-C331A: Fwd 5'-TGATGCCTGCCAGGGCGTGCACCAGGACAC-3'	Eurofins Genomics	N/A
Primer for cloning DUSP7-C331A: Rev 5'-GTGTCCTGGTGCACGCCCTGGCAGGCATCA-3'	Eurofins Genomics	N/A
Primer for cloning DUSP7-R337A: Fwd 5'-GACCGTCACTGAGGCGCTGATGCCTGCC-3'	Eurofins Genomics	N/A
Primer for cloning DUSP7-R337A: Rev 5'-GGCAGGCATCAGCGCCTCAGTGACGGTC-3'	Eurofins Genomics	N/A
Primer for cloning DUSP7-1-181aa: Fwd 5'-GGGGACAAGTTTGTACAAAAAAGCAGGCTTCATGGGGAAAAACCAGCTCCGCGGCCCCCCAGCG-3'	Eurofins Genomics	N/A
Primer for cloning DUSP7-1-181aa: Rev 5'-GGGGACCACTTTGTACAAGAAAGCTGGGTCTCACTCTGTTTGAAACTTGTTG-3'	Eurofins Genomics	N/A
Primer for cloning DUSP7-56-181aa: Fwd 5'-GGGGACAAGTTTGTACAAAAAAGCAGGCTTCATGGGGAGCGCCGAGTGGCTGCAGG-3'	Eurofins Genomics	N/A
Primer for cloning DUSP7-56-181aa: Rev 5'-GGGGACCACTTTGTACAAGAAAGCTGGGTCTCACTCTGTTTGAAACTTGTTG-3'	Eurofins Genomics	N/A
Primer for cloning DUSP7-252-384aa: Fwd 5'-GGGGACAAGTTTGTACAAAAAAGCAGGCTTCATGGGGCTCTACCTCGGCTGCGCCAAGG-3'	Eurofins Genomics	N/A

Primer for cloning DUSP7-252-384aa: Rev 5'-GGGGACCACTTTGTACAAGAAAGCTGGGTCTCACGTCCGCTCAAAGTCCAGC-3'	Eurofins Genomics	N/A
Primer for cloning DUSP7-252-419aa: Fwd 5'-GGGGACAAGTTTGTACAAAAAAGCAGGCTTCATGGGGCTCTACCTCGGCTGCGCCAAGG-3'	Eurofins Genomics	N/A
Primer for cloning DUSP7-252-419aa: Rev 5'-GGGGACCACTTTGTACAAGAAAGCTGGGTCTCACGTGGACTCCAGCGTATTGAGTGG-3'	Eurofins Genomics	N/A
Primer for cloning DUSP7-Rescue: Fwd 5'-GTAGGCGTCGTTGAGTGAAAGATTCATCTTCTGCATCAGATA-3'	Eurofins Genomics	N/A
Primer for cloning DUSP7-Rescue: Rev 5'-TATCTGATGCAGAAGATGAATCTTTCACTCAACGACGCCTAC-3'	Eurofins Genomics	N/A
Primer for cloning ERK2-T185,Y187A: Fwd 5'-TACCAACGTGTGGCCACAGCTTCTGCCAGGAACCCTGTGTGATC-3'	Eurofins Genomics	N/A
Primer for cloning ERK2-T185,Y187A: Rev 5'-GATCACACAGGGTTCCTGGCAGAAGCTGTGGCCACACGTTGGTA-3'	Eurofins Genomics	N/A
Primer for cloning ERK2-D318N: Fwd 5'-CTCGTCACTCGGGTTGTAATACTGCTCCAGATATGG-3'	Eurofins Genomics	N/A
Primer for cloning ERK2-D318N: Rev 5'-CCATATCTGGAGCAGTATTACAACCCGAGTGACGAG-3'	Eurofins Genomics	N/A
Primer for cloning ERK2-D321N: Fwd 5'-GATGGGCTCGTTACTCGGGTCGTAATACTGCTC-3'	Eurofins Genomics	N/A
Primer for cloning ERK2-D321N: Rev 5'-GAGCAGTATTACGACCCGAGTAACGAGCCCATC-3'	Eurofins Genomics	N/A
Primer for cloning ERK2-D318,321N: Fwd 5'-CGATGGGCTCGTTACTCGGGTTGTAATACTGCTCCAGAT-3'	Eurofins Genomics	N/A
Primer for cloning ERK2-D318,321N: Rev 5'-ATCTGGAGCAGTATTACAACCCGAGTAACGAGCCCATCG-3'	Eurofins Genomics	N/A
Recombinant DNA		
DUSP7 cDNA	GenScript	Clone ID: OHu03759C; NM_001947
pDONR221-MAPK1 (original clone FLH182003.01X)	DNASU Plasmid Repository	Clone ID: HsCD00076104
pGLAP1-DUSP7	This paper	N/A
pGLAP1-DUSP7-R102A	This paper	N/A

pGLAP1-DUSP7-R103A	This paper	N/A
pGLAP1-DUSP7-R102,103A	This paper	N/A
pGLAP1-DUSP7-C331A	This paper	N/A
pGLAP1-DUSP7-R337A	This paper	N/A
pGLAP1-DUSP7-1-181aa	This paper	N/A
pGLAP1-DUSP7-56-181aa	This paper	N/A
pGLAP1-DUSP7-252-384aa	This paper	N/A
pGLAP1-DUSP7-252-419aa	This paper	N/A
pGLAP1-DUSP7-Rescue	This paper	N/A
pGLAP1-DUSP7-Rescue-C331A	This paper	N/A
pGLAP1-DUSP7-Rescue-R337A	This paper	N/A
pCS2-HA-DUSP7	This paper	N/A
pCS2-Flag-DUSP7	This paper	N/A
pCS2-Flag-DUSP7-C331A	This paper	N/A
pCS2-Flag-DUSP7-R337A	This paper	N/A
pGBioID2-DUSP7	This paper	N/A
pGLAP1-ERK2	This paper	N/A
pGLAP1-ERK2-2A(T185,Y187A)	This paper	N/A
pCS2-HA-ERK2	This paper	N/A
pCS2-Flag-ERK2	This paper	N/A
pCS2-Flag-ERK2-2A(T185,Y187A)	This paper	N/A
pCS2-HA-ERK2-D318N	This paper	N/A
pCS2-HA-ERK2-D321N	This paper	N/A
pCS2-HA-ERK2-D318,321N	This paper	N/A
pCS2-Flag-GFP	This paper	N/A
pCS2-HA-GFP	This paper	N/A
Software and Algorithms		
GraphPad Prism 5	GraphPad	RRID: SCR_002798
Adobe Photoshop	Adobe	http://shop.adobe.com/store/adbehap/DisplayHomePage
Serial Cloner 2.6	SerialBasic	http://serialbasics.free.fr/Serial_Cloner.html
ImageJ	NIH ImageJ	https://imagej.nih.gov/ij/index.html
Biorender	Biorender	RRID: SCR_018361
R Scripts for Mass Spectrometry Analyses	Torres Lab	GitHub: https://github.com/uclatorreslab/MassSpecAnalysis

Table S3–S4. List of all protein identifications from LAP (Table S3) and BioID2 (Table S4) purifications, including protein accession number, number of distinct peptides assigned for each protein, and derived protein identification probability.

Table S5–S6. List of single-peptide identifications of proteins from Figure 1A (Table S5) and Figure 1B (Table S6), including annotated mass-labeled MS/MS spectra.

DETAILED EXPERIMENTAL PROCEDURES

Cell Synchronization, Transfection and Inhibitors Treatment

For G1/S arrest and release, cells were arrested with 2 mM thymidine (Sigma-Aldrich) for 16–18 hours and washed three times with PBS, two times with complete medium before being released into fresh medium. For mitotic arrest, cells were arrested with 100 nM Taxol (Sigma-Aldrich) or 330 nM nocodazole (Sigma-Aldrich) for 18 hours. For metaphase arrest, cells were arrested with 10 μM MG132 (Millipore Sigma) for one-hour post thymidine release. For transient transfection, HeLa cells were transfected with the plasmids generated above with FuGENE HD (Promega) for 24–48 hours before harvesting or fixation. For siRNA experiments, cells were transfected with Silencer Select Validated siRNA from Thermo Fisher Scientific targeting DUSP7 (siRNA ID: s4381) or ERK2 (siRNA ID: s11138) at 20 μM using Lipofectamine RNAiMAX (Thermo Fisher Scientific) for 50–72 hours before harvesting or fixation. For MEK inhibition, cells were treated with 50 μM U0126 (Selleckchem) for 1 hour or 18 hours in the case of immunoprecipitation. For ERK2 inhibition, cells were treated with 50 μM FR 180204 (Selleckchem) for two hours.

Generation of Stable Cell Lines

pGLAP1-only/DUSP7/DUSP7-C331A/DUSP7-R337A/ERK2/ERK2-2A/DUSP7-truncations and pGBioID2-only/DUSP7 were co-transfected with pOG44 Flp-Recombinase Expression Vector (Thermo Fisher Scientific) into HeLa Flp-In T-REx cell lines by FuGENE 6 (Promega). After selecting the integrated cells with 400 μg ml^{-1} Hygromycin B (Thermo Fisher Scientific), the individual colonies were collected and continually grown for protein induction test before further protein purification experiments.

LAP/BioID2 Purifications

For LAP purifications, LAP-only and LAP-DUSP7 stable cell lines were induced with 0.1 μg ml^{-1} doxycycline (Sigma-Aldrich) and treated with 100 nM Taxol for 18 hours before being harvested and lysed. The cell lysates were subjected to tandem affinity purification by incubating with anti-GFP antibody beads; the bound eluates were incubated with S-protein Agarose (Millipore Sigma). The final eluates were resolved on a 4%–20% gradient SDS PAGE gel (Bio-Rad); the gel was excised and prepared for LC-MS/MS analysis. For BioID2 purifications, BioID2-only and BioID2-DUSP7 stable cell lines were washed with PBS and DMEM/Ham's F-12 before being shifted into DMEM/Ham's F-12 supplemented with 10% streptavidin Dynabeads (Thermo Fisher Scientific) treated FBS (FBS was incubated with Dynabeads at 4°C overnight and the Dynabeads were removed with a magnetic stand the following day). The cells were induced with 0.1 μg ml^{-1} doxycycline and treated with 100 nM Taxol and 50 μM Biotin (Sigma-Aldrich) for 16 hours before being lysed in lysis buffer (50 mM Tris-HCl pH 7.5, 150 mM NaCl, 1 mM EDTA, 1 mM EGTA, 1% Triton-X-100, 0.1% SDS, protease inhibitor cocktail) for 1 hour at 4°C with gentle rotation. The cell lysates were centrifuged at 15,000 rpm for 15 minutes and transferred to TLA-100.3 tubes (Beckman Coulter) for a second high speed centrifuge at 45,000 rpm for 1 hour at 4°C. The supernatants were incubated with Dynabeads at 4°C overnight with gentle rotation. The beads were washed twice with 2% SDS, one time with WB1 (0.1% sodium deoxycholate, 1% Triton X-100, 500 mM NaCl, 1 mM EDTA, 50 mM HEPES), one time with WB2 (250 mM LiCl, 0.5% deoxycholate, 1 mM EDTA, 10 mM Tris-HCl pH 8.0), and a final wash with 50 mM Tris-HCl pH 7.5 before being resuspended in elution buffer (50 mM triethylammonium bicarbonate, 12 mM sodium lauroyl sarcosine, 0.5% sodium deoxycholate). The resuspended beads were subjected to on-bead digestion and LC-MS/MS analysis.

In Gel Digestion and In Solution Tryptic Digestion

For in gel digestion, the excised gels were dehydrated in 100% acetonitrile for 30 minutes and incubated with 5 mM tris(2-carboxyethyl) phosphine (TCEP) in 50 mM NH_4HCO_3 at 37°C for 30 minutes. The gels then were incubated with 55 mM iodoacetamide in 50 mM NH_4HCO_3 in the dark at room temperature for 30 minutes, and the liquid was discarded. The gels were dehydrated in 100% acetonitrile for 30 minutes,

incubated with 50 mM NH_4HCO_3 for 15 minutes, and dehydrated again in 100% acetonitrile for 15 minutes. The liquid was discarded and the gels were incubated with 5 ng μl^{-1} Sequencing Grade Modified Trypsin (Promega) (prepared on ice in 25 mM NH_4HCO_3 and 0.1% sodium deoxycholate) on ice for 45 minutes followed by incubation with 25 mM NH_4HCO_3 at 37°C overnight. For peptides extractions, 50% acetonitrile was added to the samples and the samples were sonicated for 15 minutes followed by a second sonication in new tubes. A third time extraction was performed with 100% acetonitrile for 20 minutes. The samples were then lyophilized by SpeedVac (ThermoFisher Scientific), reconstituted in water, and vortexed with ethyl acetate plus 0.5% trifluoroacetic acid (TFA) at 1:1 (volume:volume) ratio for five minutes. The samples were centrifuged at 16,000 x g for five minutes at room temperature and the supernatant was removed. The pellets were proceeded to C18 StageTips (ThermoFisher Scientific) desalting as described previously (50). For in solution digestion, streptavidin Dynabeads in elution buffer were boiled to 95°C for 10 minutes and then sonicated for ten minutes to denature proteins. Denatured proteins were treated with 5 mM TCEP at 37°C for 30 minutes to reduce disulfide bonds. The protein solutions were treated with 10 mM chloroacetamide and incubated in the dark for 30 minutes at room temperature for protein alkylation, before being diluted five-fold with 50 mM triethylammonium bicarbonate (TEAB) and incubated with trypsin (prepared in 50 mM TEAB and used at 1:100 (mass:mass) ratio) at 37°C for 4 hours. Target proteins were treated again with trypsin at the same concentration at 37°C overnight. The samples were vortexed with ethyl acetate plus 1% trifluoroacetic acid (TFA) at 1:1 (volume:volume) ratio for five minutes. The samples were centrifuged at 16,000 x g for five minutes at room temperature and the supernatant was removed. The pellets were then lyophilized by SpeedVac and desalted on C18 StageTips as described previously (50).

Nano-liquid Chromatography with Tandem Mass Spectrometry (LC-MS/MS) Analysis
A Q Exactive Plus Orbitrap (ThermoFisher Scientific) integrated with an Eksigent 2D nano-LC instrument was used for nano-LC-MS/MS with collision-induced dissociation. Online peptide chromatography was performed on a laser-pulled reverse-phase column, 75 µm x 200 mm, containing 5-µm C18 resin with 300-Å pores (ThermoFisher Scientific). A nanospay ion source (ThermoFisher Scientific) was used for electrospray ionization using the following conditions: capillary temperature- 200° C, tube lens- 110 V, and spray voltage- 2.3 kV. The loading and separation flow rate for reverse-phase chromatography was 500 nl min^{-1} in buffer A (0.1% formic acid, 2% acetonitrile) and buffer B (0.1% formic acid, 98% acetonitrile). Sample peptides were loaded onto the column over thirty minutes and were resolved by a 0–80% buffer B gradient for 174 minutes. The Q Exactive Plus Orbitrap was operated in data-dependent mode with a 180 minutes full precursor scan time at high resolution (70,000 at m z^{-1} 400) from 350-1,700 m z^{-1} and 10 MS/MS fragmentation scans at low resolution in the linear trap using charge-state screening excluding both unassigned and +1 charge ions. The collision-induced dissociation was performed with an intensity threshold of 500 counts and a collision energy of 40%. For dynamic exclusion, a repeat count of 1 was applied, and the exclusion duration was set to 15 seconds.

LC-MS/MS Experimental Design and Statistical Rationale
Control (LAP-only and BioID2-only) and experimental (LAP-DUSP7 and BioID2-DUSP7) purifications were performed once each to identify DUSP7 protein-protein and protein proximity associations. With the comparison between control and experimental purifications, proteins identified in the controls were presumed as potential non-specific associations. Mascot (v2.4; Matrix Science, Boston, MA) was used for database searches of the acquired spectra as described previously (46). The UniProt human database (release date March 27, 2018 for LAP purification and release date August 16, 2018 for BioID2 purification) was used with 21,044 entries actually searched, and the following search parameters were applied: trypsin digestion for LAP purification and semiTrypsin for BioID2 purification allowing up to 2 missed cleavages; carbamidomethyl on cysteine as a fixed modification; acetyl of lysine and protein N-term, deamidated of asparagine and glutamine, and oxidation of methionine as the variable modifications for LAP purification; acetyl of lysine and protein N-term, deamidated of asparagine and glutamine, oxidation of methionine, and phospho of seine, threonine and tyrosine as the variable modifications for

BioID2 purification; 10-ppm peptide mass tolerance; and 0.5-Da fragment mass tolerance. The reverse UniProt human database (with the search parameters listed above), as the decoy database, was used to obtain an overall 5% peptide false discovery rate (FDR), which accounts for total false positives and false negatives. Peptides that surpassed an expectation cut-off score of 20 were accepted. All raw mass spectrometry files are uploaded to the UCSD Center for Computational Mass Spectrometry MassIVE datasets ftp://massive.ucsd.edu/MSV000085629/. In-house R scripts were coded to analyze peptides that met the above criteria with information about their corresponding identified protein. All R scripts used in this study can be freely accessed at GitHub https://github.com/uclatorreslab/MassSpecAnalysis. A pseudo qualitative/quantitative approach was applied to increase precision and reduce error. Proteins identified in experimental purifications but not present in control purifications were further analyzed, whereas proteins identified in both the control and experimental purifications were assayed for significance. To handle proteins shared between experimental and control purifications, but only identified in less frequency, we measured the relative fold change or mean difference in a quantitative manner. The Exponentially Modified Protein Abundance Index (emPAI) was used to compare quantification between purifications (51). emPAI offers approximate relative quantitation of the proteins in a mixture based on protein coverage by the peptide matches in a database search result and can be calculated using the following equation (51).

$$emPAI = 10^{\frac{N_{Observed}}{N_{Observable}}} - 1$$

Where $N_{Observed}$ is the number of experimentally observed peptides and $N_{Observable}$ is the calculated number of observable peptides for each protein (51). Using emPAI as a relative quantification score, we calculated the mean difference (the mean emPAI for a certain protein across test replicates minus the mean emPAI for the same protein across control purifications). Using resampling in a paired manner, we then estimated the distribution of the mean difference for shared proteins between experimental and control purifications. Resampling involved calculating the mean difference many times under rearrangement of labels to recreate or estimate the normal distribution. We performed ten thousand simulations per mean difference, resulting in normal distributions of mean difference between values of proteins identified in the experimental and the control. Using this distribution, we related each individual mean difference to the mean difference observed in the overall population in order to get a relative idea of what might be significantly higher in value compared to the control, when taking what is observed in the entire population. Values that were outside of the 95% confidence interval of the mean difference and higher in the experimental than in the control were then considered for further analysis.

Immunoprecipitations, *In Vitro* Binding Assays, and Immunoblot Analyses

For immunoprecipitations, LAP-tagged inducible stable cell lines were expressed with 0.1 μg ml^{-1} doxycycline (Sigma-Aldrich) for 18 hours. The induced stable cell lines or transiently transfected HeLa cells were lysed in LAP200 lysis buffer (50 mM Hepes pH 7.4, 200 mM KCl, 1 mM EGTA, 1 mM $MgCl_2$, 10% glycerol) plus 0.05% NP-40, 0.5 mM DTT and protease inhibitor cocktail (Thermo Fisher Scientific). The cell lysates were incubated with S-protein Agarose (Millipore Sigma) at 4°C for two hours. S-protein Agarose was then washed three times with LAP100 buffer (50 mM Hepes pH 7.4, 100 mM KCl, 1 mM EGTA, 1 mM $MgCl_2$, 10% glycerol) plus 0.05% NP-40 and 0.5 mM DTT. For *in vitro* binding assays, Flag-tagged GFP/DUSP7/DUSP7-C331A/DUSP7-R337A/ERK2/ERK2-2A and HA-tagged GFP/DUSP7/ERK2/ERK2-D318N/ERK2-D321N/ERK2-D318,321N were expressed in a Quick Coupled Transcription/Translation System (Promega), incubated together with Anti-FLAG M2 (Sigma-Aldrich) or Anti-HA (MBL) magnetic beads at 4°C for 1.5 hours. The bound beads were washed three times with LAP200 buffer plus 0.05% NP-40 and 0.5 mM DTT. The samples were resolved on a 4%–20% gradient SDS PAGE gel (Bio-Rad) and transferred to a PVDF membrane (EMD Millipore). Membranes were incubated with primary antibodies in blocking buffer (PBS, 0.5% BSA, 0.05% Tween-20, 0.02% SDS, 0.05% Proclin) at 4°C overnight, then washed three times (five minutes each) with PBST (PBS, 0.1% Tween-20), and incubated with secondary antibodies conjugated to IRDye 680RD or IRDye 800CW at room temperature for 30 minutes. Western blots were scanned and analyzed on a LI-COR

Odyssey Imager (LI-COR Biotechnology). The relative intensity of western blot bands were analyzed with ImageJ.

Immunofluorescence

For immunofluorescence microscopy, HeLa, U2OS or HCT116 cells were fixed with 4% paraformaldehyde, permeabilized with 0.2% Triton X-100/PBS, and blocked with IF buffer (PBS, 5% Fish Gelatin, 0.1% TritonX-100) before being incubated with 0.5 mg ml^{-1} Hoechst 33342 and the indicated primary antibodies in IF buffer at room temperature for one hour. Cells were then washed with PBS three times (five minutes each) and incubated with secondary antibodies in IF buffer for 30 minutes. After a final wash, the coverslips were mounted with ProLong Gold Antifade mounting solution (Invitrogen) on glass slides. Images were captured with a Leica DMI6000 microscope (Leica DFC360 FX Camera, 63x/1.40–0.60 NA oil objective, Leica AF6000 software), deconvolved with Leica Application Suite 3D Deconvolution software, and exported as TIFF files. For live-cell time-lapse microscopy, HCT116 GFP-H2B cells were imaged live six hours post thymidine release for 18 hours with an ImageXpress XL imaging system (Molecular Devices) at 37°C in 5% CO_2 using a 20x air objective. Images were captured every five minutes with bright field and FITC channel, and converted to AVI movies with ImageJ at one frame per second.

Quantification and Statistical Analyses

For fixed cell immunofluorescence microscopy quantification, for each condition three independent experiments were performed with 100 cells counted per experiment (n=300), except for Figure 2G where three independent experiments were performed with 300 cells counted per experiment (n=900). For live cell time-lapse microscopy quantification, for each condition three independent experiments were performed with 30 cells counted per experiment (n=90). The data were analyzed using unpaired Student's t test in Figure 2D, 2G, 2K, 3D, 3H, 4C, 4E, S3E, S3H, S4E, S4F, S4H, S4I, S4K, S4L, S5B, S5D, S5F, S5J, S5L, S5N, S6A and S6B; paired Student's t test in Figures S4B, S4C, S4G and S4J. The Livak-Schmittgen method ($2^{-\Delta\Delta Cq}$) was used to analyze qPCR data. Data is judged to be statistically significant when $P<0.05$. More specifically, asterisks indicate statistical significance as * $P<0.05$, ** $P<0.01$, *** $P<0.001$. All statistical data were presented as the mean±s.d. All statistical figures were generated with GraphPad Prism 5.
